# Epipolythiodiketopiperazines from the Marine Derived Fungus *Dichotomomyces cejpii* with NF-κB Inhibitory Potential

**DOI:** 10.3390/md13084949

**Published:** 2015-08-06

**Authors:** Henrik Harms, Barbora Orlikova, Seungwon Ji, Damun Nesaei-Mosaferan, Gabriele M. König, Marc Diederich

**Affiliations:** 1 Institute for Pharmaceutical Biology, University of Bonn, Nussallee 6, Bonn D-53115, Germany; E-Mails: henrik-harms@gmx.de (H.H.); damun1363@hotmail.de (D.N.-M.); 2 Department of Pharmacy, College of Pharmacy, Seoul National University, 1 Gwanak-ro, Gwanak-gu, Seoul 151-742, Korea; E-Mails: barbora.orlikova@lbmcc.lu (B.O.); jsw0525@snu.ac.kr (S.J.); 3 Laboratoire de Biologie Moléculaire et Cellulaire du Cancer (LBMCC), Hôpital Kirchberg, 9 rue Edward Steichen, Luxembourg L-2540, Luxembourg

**Keywords:** Ascomycete, *Dichotomomyces*, gliotoxin, NF-κB, leukemia

## Abstract

The Ascomycota *Dichotomomyces cejpii* was isolated from the marine sponge *Callyspongia cf. C. flammea.* A new gliotoxin derivative, 6-acetylmonodethiogliotoxin (**1**) was obtained from fungal extracts. Compounds **2** and **3**, methylthio-gliotoxin derivatives were formerly only known as semi-synthetic compounds and are here described as natural products. Additionally the polyketide heveadride (**4**) was isolated. Compounds **1**, **2** and **4** dose-dependently down-regulated TNFα-induced NF-κB activity in human chronic myeloid leukemia cells with IC_50s_ of 38.5 ± 1.2 µM, 65.7 ± 2.0 µM and 82.7 ± 11.3 µM, respectively. The molecular mechanism was studied with the most potent compound **1** and results indicate downstream inhibitory effects targeting binding of NF-κB to DNA. Compound **1** thus demonstrates potential of epimonothiodiketopiperazine-derived compounds for the development of NF-κB inhibitors.

## 1. Introduction

The secondary metabolism of fungi gives rise to chemically most diverse structures, some of which have proven useful in drug development. A promising source for new secondary metabolites are organisms isolated from special habitats, like marine-derived fungi and bacteria [1]. Accordingly, fungal secondary metabolites have gained popularity due to their newly discovered pharmaceutical potential as anti-inflammatory, cytotoxic or cytostatic agents.

A number of gliotoxin derivatives have been isolated inter alia from fungus *Penicillium* sp. strain JMF034, from Japanese deep-sea sediments. These marine-derived drug candidates display epigenetic and anti-cancer activities against P388 murine leukemia cells. Compounds containing a disulfide bond including gliotoxin G, 5a,6-didehydrogliotoxin and gliotoxin showed potent *in vitro* inhibitory activity against the recombinant H3K9 histone methyl transferase G9a. The presence of a disulfide bond is usually accompanied by distinct toxic effects that limit the therapeutic usage of this compound group. Here, the Ascomycete *Dichotomomyces cejpii*, isolated from the marine sponge *Callyspongia cf*. *C. flammea*, allowed isolation of new gliotoxin derivatives devoid of a disulfide bridge. Along with the likewise isolated polyketide heveadride these compounds were investigated as anti-inflammatory, cytotoxic or cytostatic agents [2].

Several gliotoxins have been identified as potent inhibitors of cancer associated inflammatory signaling pathways [3]. One of the crucial pathways linking cancer and inflammation is nuclear factor kappa B (NF-κB). Growing evidence indicates the involvement of this transcription factor in many inflammatory diseases and cancer progression. NF-κB became a promising therapeutic target of both hematological malignancies and solid tumors [4]. Many risk factors related to life style activate inflammatory pathways via NF-κB. Aberrantly activated NF-κB leads to expression of target genes involved in all steps of tumorigenesis. Moreover, constitutively active NF-κB serves as a useful prognostic indicator.

NF-κB is an inducible factor that acts as a dimer of pair-wise combinations of proteins from the Rel family [5]. This family comprises five members, RelA (p65), Rel (c-Rel), RelB, NF-κB1 (p105/p50) and NF-κB2 (p100/p52), which require dimerization prior to activation. Preferential dimer-binding is conditional for DNA affinity. In comparison to most eukaryotic transcription factors, the p50/p65 heterodimer presents a particularly high DNA affinity, which is why this dimer interacts with DNA more frequently than other NF-κB dimers [6]. Upon pathway activation through ligand binding, the natural inhibitor of kappa B (IκB), which is sequestering the inactive p50/p65 dimer in the cytoplasm, is phosphorylated, ubiquitinated and degraded by the proteasome. Active dimer translocates to the nucleus, binds to corresponding DNA sequences in promoter or enhancer regions and is responsible for the expression of pro-inflammatory genes [7].

In the present study we investigated the inhibitory effect of newly isolated natural epipolythiodiketopiperazines on anti-proliferative mechanisms via TNFα-induced p50/p65 NF-κB activity to understand cytostatic effects of these marine-derived drug candidates.

## 2. Results and Discussion

### 2.1. Structure Elucidation

Compound **1** was obtained from the marine sponge-derived fungus *D. cejpii* and its structure was elucidated via intensive analysis of spectroscopic data. A Ultraviolet (UV) maximum at 262 nm evidenced the presence of a conjugated π-π* system. A broad Infrared (IR) absorption at 3425 cm^−1^ pointed toward a hydroxyl group, while a strong IR absorption at 1722 cm^−1^, arising from C=O stretching frequencies, indicated an ester moiety (Figures S1.1 and S1.2). The molecular formula of compound **1** was deduced from the results of an accurate mass measurement using high-resolution electrospray ionisation mass spectrometry (HRESIMS), *m*/*z* = 359.0672 [M + Na]^+^ as C_15_H_16_N_2_O_5_S, implying nine degrees of unsaturation (Figure S1.11). The ^13^C Nuclear Magnetic Resonance (NMR) and Distortionless Enhancement by Polarization Transfer-135 (DEPT-135) spectra denoted the presence of 15 resonances for two methyl groups, two sp^3^ methylene groups, three sp^2^ methine, two sp^3^ methine, and six quaternary carbons in the molecule (Table 1, Figures S1.3–S1.9).

**Table 1:** NMR Spectroscopic Data of Compounds **1**–**3** in acetone-d*_6_* (^1^H: 300 MHz; ^13^C: 75 MHz).

Position	Compound 1		Compound 2		Compound 3	
	δ_C/N_, Type	δ_H_ (*J* in Hz)	δ_C/N_, Type	δ_H_ (*J* in Hz)	δ_C/N_, Type	δ_H_ (*J* in Hz)
1	174.2, C	-	166.8, C	-	166.3, C	-
2	N	-	N	-	N	-
3	82.5, C	-	74.5, C	-	73.2, C	-
3a	58.0, CH_2_	3a′: 4.30, dd (5.9, 13.2) 3a″: 4.17, dd (5.9, 13.2)	64.3, CH_2_	3a′: 4.20, dd (5.9, 11.3) 3a″: 3.72, dd (5.9, 11.3)	64.8, CH_2_	3a′: 4.42, dd (4.0, 12.1) 3a″: 3.95, dd (4.0, 12.1)
4	171.3, C	-	164.9, C	-	162.9, C	-
5	N	-	N	-	N	-
5a	61.6, CH	4.57, br d (13.5)	66.3, CH	5.10, br d (13.5)	142.4, C	-
6	74.9, CH	5.79, br d (13.5)	75.7, CH	6.17, br d (13.5)	118.3, CH	8.02, d (7.3)
7	127.1, CH	5.53, br d (9.9)	128.2, CH	5.57, br d (9.9)	128.3, CH	7.30, t (7.3)
8	125.7, CH	6.01, m	126.2, CH	6.01, m	126.3, CH	7.19, t (7.3)
9	119.5, CH	6.03, br s	120.0, CH	6.03, br s	126.2, CH	7.39, d (7.3)
9a	137.4, C	-	135.8, C	-	130.3, C	-
10	29.2, CH_2_	10′: 3.45, d (18.3) 10″: 2.98, d (18.3)	40.4, CH_2_	10′: 3.09, d (15.7) 10″: 2.79, d (15.7)	40.0, CH_2_	10′: 3.59, d (16.8) 10″: 3.51, d (16.8)
10a	80.4, C	-	73.6, C	-	71.6, C	-
11	27.8, CH_3_	2.98, s	14.9, CH_3_	2.25, s	14.4, CH_3_	2.22, s
12	170.5, C	-	28.7, CH_3_	3.03, s	28.9, CH_3_	3.15, s
13	21.2, CH_3_	2.06, s	12.7, CH_3_	2.18, s	13.5, CH_3_	2.32, s
14			170.6, C	-		
15			21.3, CH_3_	2.03, s		
3a-OH	-	4.74, br t (5.9)	-	4.50, br t (5.9)	4.62, br t (4.0)	

The first substructure was deciphered as a cyclohexadiene by interpreting ^1^H-^1^H Correlation Spectroscopy (COSY) correlations from H-5a through to H-9, and Heteronuclear Multiple Bond Correlation (HMBC) cross peaks between the resonances for H-9 and C-9a, C-5a, C-6, C-7 and C-8. The two conjugated carbon-carbon double bounds within this substructure could be assigned as ∆^7,8^ and ∆^9,9a^ due to the ^13^C NMR chemical shifts of the respective carbon atoms (127.1/125.7, 119.5/137.4 ppm). The methine group CH-6 resonated at δ_H_ 5.79 and δ_C_ 74.9, indicating a substitution with oxygen at this site. Due to its ^13^C NMR chemical shift (δ_C_ 170.5) C-12 was part of a carbonyl group. HMBC correlations arising from the resonance of CH_3_-13 to C-12, as well as to CH-6 established that an acetyl group was linked via the oxygen atom at CH-6. An extension of this first substructure was possible taking HMBC cross peaks between the resonances for the methylene group CH_2_-10 to C-9a on one side, and to C-10a and C-1 on the other side into account. The ^13^C NMR chemical shifts of C-10a (δ_C_ 80.4) and of the protons and carbon atoms of the methine group CH-5a (δ_H_ 4.57, δ_C_ 61.6), indicated a substitution with a heteroatom at these sites, whereas C-1 was a carbonyl (δ_C_ 174.2). CH_3_-11 resonating at 2.98 and 27.8 ppm, respectively had to be linked to nitrogen. HMBC cross peaks between the resonances for this methyl group to the carbonyl carbon atom C-1 thus revealed an amide bond. A second amide was obvious when considering the ^13^C NMR chemical shift (δ_C_ 171.3) for C-4. ^1^H NMR chemical shifts of δ_H_ 4.30 and 4.17 for the geminal methylene protons 3a′ and 3a″ indicated the presence of a CH_2_OH moiety. Regarding HMBC cross peaks between the resonances for these methylene group protons CH_2_-3a to carbons C-3 and C-4, and of CH_3_-11 to C-1 and C-3, the second substructure from C-1 through to C-4 (including C-11) could be deduced, which was linked via N-5 to the first substructure.

Thus compound **1** is a diketopiperazine with a dihydro-indoline moiety. The molecular formula indicated the presence of one single sulphur atom within the molecule. As this point of the structure elucidation also two double bond equivalents were still to be accounted for. In accordance with the MS data, the sulphur atom was thus assigned to form a sulphur bridge between carbon C-10a and C-3, due to the ^13^C NMR chemical shift of the respective carbons (C-3: 82.5 ppm, C-10a: 80.4 ppm). This is comparable to the disulfide bridge in the related compound gliotoxin (C-10a and C-3: δ_C_ 77.3 and 77.5, respectively) [8,9,10]. Overall, this structural arrangement of **1** is consistent with the established structures of gliotoxin-type metabolites and with recent biosynthetic evidence for this class of compounds [11,12].

The relative configuration was deduced on the basis of a Nuclear Overhauser Effect Spectroscopy (NOESY) experiment and the magnitude of significant ^1^H-^1^H coupling constants (Figure S1.10). The coupling constant of *J*_H-5a/H-6_ = 13.5 Hz indicated that both protons are orientated 180° to each other, *i.e.*, H-6 is axial and α-orientated whereas the axial H-5 is β-orientated. NOESY correlations between H-3a″, H-5a and H-10′ indicated that these protons are located on the same side, of the molecule, whereas NOESY correlations between H-3a′, H-6 and H-10″ proved the opposite orientation for these substituents. This again confirmed that proton H-5a and H-6 are trans orientated to each other. The configuration at the quaternary carbon C-10a could not be determined. We assume it to be as that of the co-occurring 6-acetylbisdethiobis(methylthio)gliotoxin (**2**) with the same basic structural arrangement [13,14]. Furthermore the fungus *D. cejpii* is a known producer of gliotoxin for which the absolute configuration had been determined and confirmed by biosynthetic evidence, the latter demonstrating the necessity of this configuration [8,10,11,13]. Fungal metabolite **1** is thus a naturally occurring gliotoxin derivative with the untypical feature of a single sulphur atom bridge. We suggest the trivial name 6-acetylmonodethiogliotoxin for **1**.

Aside from **1**, a further gliotoxin derivative, an acetylated dithiodiketopiperazine with two methylthio substituents, *i.e.*, 6-acetylbisdethiobis(methylthio)gliotoxin (**2**) was isolated for the first time as a natural product. The structure of **2** was elucidated on the basis of detailed ^1^H and ^13^C NMR data (Figures S2.3–S2.10) and comparison with literature values of the semi-synthetically obtained compound [14] (Table 1). ^1^H NMR values of **2** match very well with literature data. There were slight differences, however between the here observed ^13^C NMR resonances for carbon C-3 and literature values (+3.6 ppm). These differences may be explained by the fact that reported ^13^C NMR data had been determined only indirectly via HMBC correlations. Furthermore, it is known that NMR data of gliotoxin and its derivatives are strongly influenced by temperature and solvent [10]. Mass spectrometric data are according to the here shown structure and literature data.

**Figure 1:**
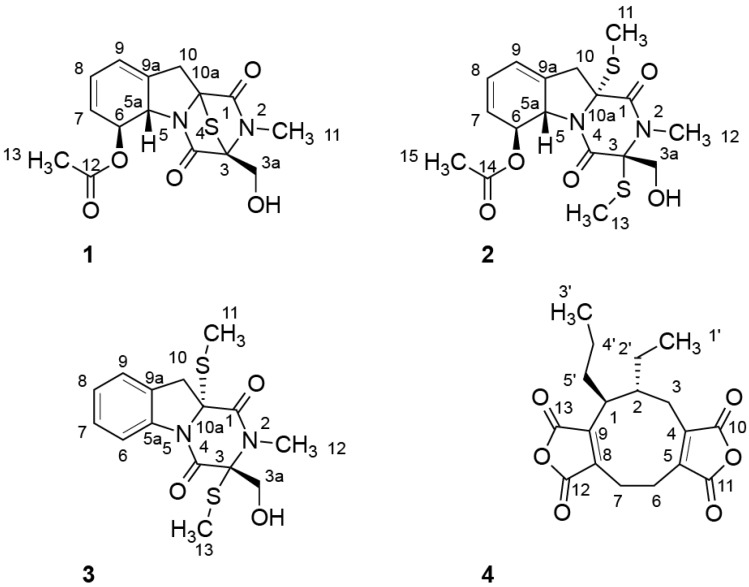
Structures of 6-acetylmonodethiogliotoxin (**1**), 6-acetylbisdethiobis(methylthio) gliotoxin (**2**), 5a,6-anhydrobisdethiobis(methylthio)gliotoxin (**3**) and heveadride (**4**) isolated from the marine-derived fungus *Dichotomomyces cejpii*.

5a,6-Anhydrobisdethiobis(methylthio)gliotoxin (**3**), an aromatic gliotoxin derivative similar to compound **2**, could also be isolated for the first time as a natural product during this study. The structure of **3** was elucidated on the basis of detailed ^1^H and ^13^C NMR data (Figures S3.1–S3.8) and comparison with literature values for the formerly semi-synthetically produced compound [15,16]. Comparison of NMR, MS and optical rotation data with literature values led to the identification of compound (**4**) as heveadride [15,16], Structures of compound **1**–**4** are shown in Figure 1.

### 2.2. Bioactivity

#### 2.2.1. Epipolythiodiketopiperazines Dose-Dependently Inhibit TNF Alpha-Induced NF-κB Activation

**Figure 2:**
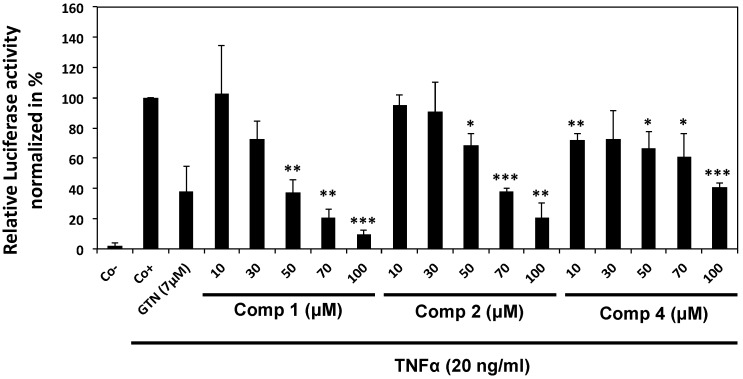
Inhibition of TNF alpha-induced NF-κB activation by pre-treatment with epipolythiodiketopiperazines. K562 cells were transiently transfected with both, firefly luciferase vector (NF-κB pGL4) and ph-RG-tk *Renilla* plasmid for 24 h. After transfection, K562 cells were treated with compound **1** (6-acetylmonodethiogliotoxin), **2** (6-acetylbisdethiobis(methylthio)gliotoxin) or **4** (heveadride) at indicated concentrations for 2 h followed by a TNFα-treatment (20 ng/mL) during 6 h. The cells were assayed for Luciferase activity. Each value is a mean ± SD of three independent experiments. Negative control (Co−) corresponds to DMSO treated cells, without TNFα activation, positive control (Co+) corresponds to DMSO treated cells activated by TNFα. Goniothalamin (GTN) at concentration 7 μM was used as a positive inhibitory control. Asterisks indicate a significant difference between untreated and 6-acetylmonodethiogliotoxin-treated cells as analyzed by *t*-test (* *p* < 0.05; ** *p* < 0.01; *** *p* < 0.001).

Compounds **1**, **2** and **4** were analyzed in order to assess their inhibitory potential on TNFα-induced NF-κB activity in K562 cells. Considering the low amount of compound **3** available, we were unable to include this compound in biological assays. We determined compounds inhibition potential on TNFα-induced activation of a luciferase gene under the control of canonical NF-κB response elements. We treated transiently transfected K562 cells at different concentrations of compounds **1**, **2** and **4**, respectively, followed by the stimulation with TNFα. Results show that TNFα-induced NF-κB reporter gene activity was dose-dependently decreased by compounds **1**, **2** and **4** compared to control with IC_50_values of 38.5 ± 1.2 µM (compound **1**), 65.7 ± 2.0 µM (compound **2**) and 82.7 ± 11.3 µM (compound **4**) (Figure 2). IC_50_ values were calculated using XY scatter dependency chart. The 50% inhibition activity on NF-κB expression was established by using the best fitting model and calculated by trend formulas. The average value of at least 3 independent experiments was applied. Molecular mechanism of compound **1** (6-acetylmonodethiogliotoxin) was investigated in more detail.

#### 2.2.2. 6-Acetylmonodethiogliotoxin Down-Regulates the Expression of NF-κB Target Genes

**Figure 3:**
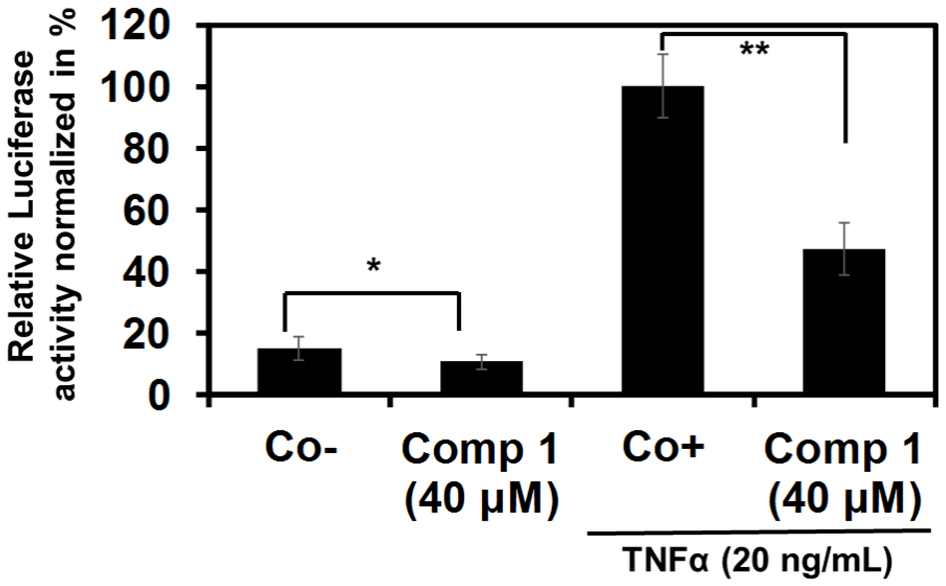
6-acetylmonodethiogliotoxin inhibits TNFα-induced NF-κB-dependent ICAM-1 gene expression. 6-acetylmonodethiogliotoxin (Compound **1**) inhibits NF-κB-dependent ICAM-1 genes expression. K562 cells were transiently transfected with ICAM-1 along with ph-RG-tk *Renilla* plasmid for 24 h. After transfection, K562 cells were treated or not with 6-acetylmonodethiogliotoxin at IC_50_ concentrations for two hours followed by a TNFα-treatment (20 ng/mL) during 6 h. The cells were assayed for Luciferase activity. Each value is a mean ± SD of three determinations. Asterisks indicate a significant difference compared to control positive as analyzed by *t*-test (* *p* < 0.05; ** *p* < 0.01). Negative control (Co−) corresponds to transfected and DMSO only treated cells, without TNFα activation, positive control (Co+) corresponds to transfected and DMSO treated cells activated by TNFα.

NF-κB signaling results in activation of a large battery of target genes. Many of these genes have been associated with different steps of tumorigenesis [17]. In order to further validate the previously observed inhibition of NF-kB reporter gene activity we investigated whether 6-acetylmonodethiogliotoxin affects ICAM-1 gene transcription. K562 cells were transiently transfected with ICAM-1 plasmid followed by treatment with 6-acetylmonodethiogliotoxin at IC_50_ concentration, and then exposed to TNFα. Our results show that TNFα induced ICAM-1 promoter-driven reporter gene activity and 6-acetylmonodethiogliotoxin significantly inhibited this induction by 53% compared to control (Figure 3).

#### 2.2.3. 6-Acetylmonodethiogliotoxin Mediated Downstream Inhibition of NF-κB Signaling by Preventing Binding of p65 to DNA

**Figure 4:**
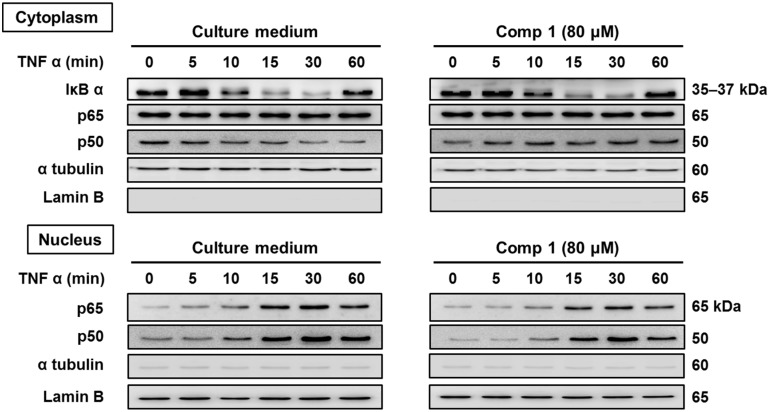
Effect of 6-acetylmonodethiogliotoxin on the degradation of IκBα and translocation of p65 and p50 to the nucleus. Jurkat cells were pre-treated with 6-acetylmonodethiogliotoxin (Compound **1**) at 80 μM for 2 h followed by activation with TNFα (20 ng/mL) for indicated time periods. Cytoplasmic and nuclear extracts were tested for IκBα, pIκBα, p50 and p65. Protein loading and purity of extracts were verified by lamin B and α-tubulin Western blots.

We further analyzed the molecular mechanism underlying the inhibition potential of 6-acetylmonodethiogliotoxin on TNFα-induced NF-κB activation. Here, we focused on degradation of IκBα, the natural inhibitor of NF-κB as well as on translocation of p50 and p65 subunits to the nucleus. As shown in Figure 4, 6-acetylmonodethiogliotoxin did neither prevent IκBα degradation, nor p50/p65 nuclear translocation. These results indicate that 6-acetylmonodethiogliotoxin mediated downstream inhibition of NF-κB pathway. As both subunits p50 and p65 translocated to the nucleus, 6-acetylmonodethiogliotoxin could either prevent their binding to DNA or abrogate NF-κB transcriptional activity.

**Figure 5:**
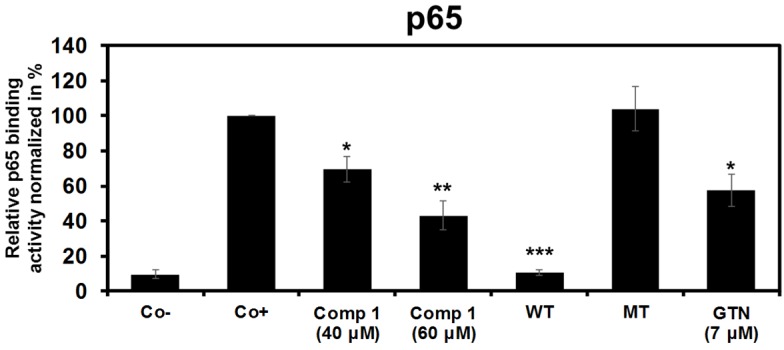
6-acetylmonodethiogliotoxin (1) reduces binding affinity of p65 to DNA in a dose dependent manner. Dose-dependent effects of 6-acetylmonodethiogliotoxin (Compound **1**) on p65 binding affinity to DNA. Jurkat cells were pre-incubated with 6-acetylmonodethiogliotoxin at indicated concentrations for 4 h and treated with TNFα (20 ng/mL) for 30 min, and then subjected to TransAM assay. Negative control (Co−) corresponds to nuclear extracts of DMSO treated cells, without TNFα activation, positive control (Co+) corresponds to nuclear extracts of DMSO treated cells activated by TNFα, wild-type (WT) and mutated type (MT) corresponds to nuclear extracts of DMSO treated cells activated by TNFα loaded on plate comprising wild-type or mutated consensus oligonucleotide, respectively. Goniothalamin (GTN) at concentration 7 μM was used as a positive inhibitory control. Shown data are mean ± SD of three independent experiments. Asterisks indicate a significant difference compared to control positive as analyzed by *t*-test (* *p* < 0.05; ** *p* < 0.01; *** *p* < 0.001).

In order to validate our hypothesis we investigated whether 6-acetylmonodethiogliotoxin is able to interfere with the binding affinity of NF-κB to its consensus response element. Figure 5 showed that 6-acetylmonodethiogliotoxin at IC_50_ concentrations significantly decreased binding affinity of p65 to the NF-κB consensus and this effect was further more pronounced at the higher concentration, whereas p50 was not affected by 6-acetylmonodethiogliotoxin treatment (data not shown). Altogether, these results provide evidence that 6-acetylmonodethiogliotoxin acts as a downstream inhibitor of NF-κB pathway altering binding of p65 to DNA.

**Figure 6:**
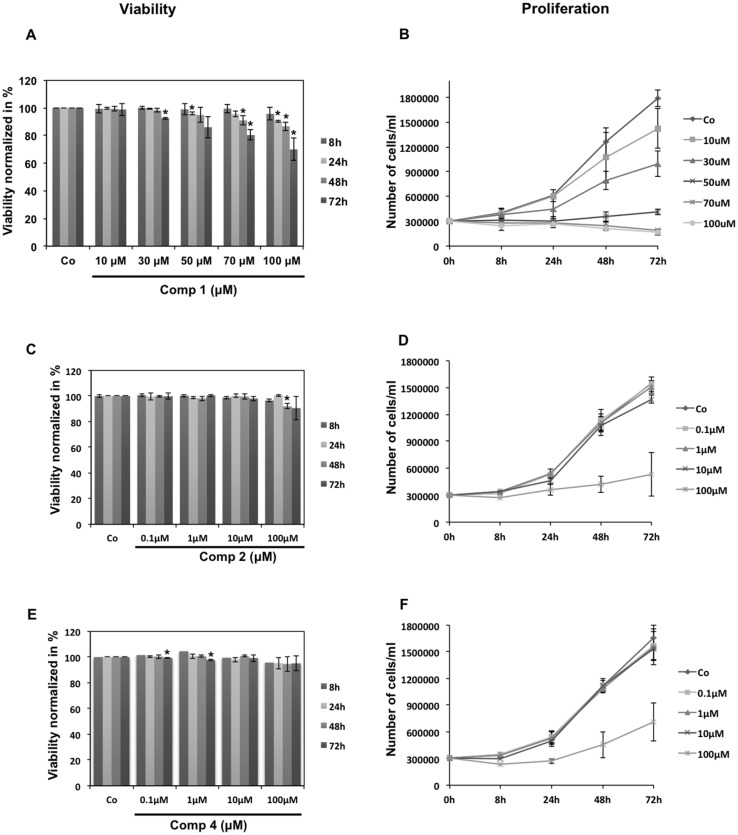
Effects of epipolythiodiketopiperazines on cancer cells viability and proliferation. Effects of compound **1** (6-acetylmonodethiogliotoxin) (**A**,**B**), compound **2** (6-acetylbisdethiobis(methylthio)gliotoxin) (**C**,**D**) and compound **4** (heveadride) (**E**,**F**) at the indicated concentrations on K562 cells viability and proliferation respectively, analyzed after 8, 24, 48 and 72 h. Control (Co) corresponds to untreated cells. Each value is a mean ± SD of three determinations. The asterisk indicates a significant difference compared to the control as analyzed by *t*-test (* *p* < 0.05).

As the NF-κB pathway is known to regulate a large battery of genes involved in cell proliferation and survival, we investigated the effects of compound **1** (6-acetylmonodethiogliotoxin) (Figure 6A,B), compound **2** (6-acetylbisdethiobis(methylthio)gliotoxin) (Figure 6C,D) and compound **4** (heveadride) (Figure 6E,F) on K562 cell viability and proliferation.

Importantly, any of the tested compounds decreased cancer cell viability after 8 h of treatment used for the investigation of NF-κB mechanisms, reflecting that down-regulation of NF-κB activation was not due to cell death induction. At later time points (24 h, 48 h and 72 h), 6-acetylmonodethiogliotoxin dose-dependently inhibited cancer cell proliferation (Figure 6B). At 50 μM, compound **1** (6-acetylmonodethiogliotoxin) induced cytostatic effects and at higher concentrations of 70 μM and 100 μM a moderate cytotoxicity was observed reaching a maximum of 20% and 30% at 72 h, respectively. These observations could be the secondary consequences of early NF-κB inhibition.

However, compound 2 (6-acetylbisdethiobis(methylthio)gliotoxin) and compound 4 (heveadride), are weaker NF-κB inhibitors compared to compound 1 (6-acetylmonodethiogliotoxin) and did not significantly decrease K562 cell viability at any concentration or measured time point (Figure 6C,E, respectively). On the other hand, 100 μM of both compounds reduced K562 proliferation compared to control. Whether these effects could be directly linked to the observed NF-κB down-regulation will be object of future studies.

### 2.3. Discussion

Our data indicate strong anti-proliferative potential on chronic myeloid leukemia cells, as 6-acetylmonodethiogliotoxin dose- and time-dependently inhibited K562 cell proliferation.

Importance of inflammation in cancer initiation and progression is nowadays well established. Many epidemiological, experimental and clinical studies strengthen the tight relationship between cancer and inflammation [18]. During past decades, NF-κB, as a crucial player in inflammatory signaling, became a popular target in cancer therapy. Abnormal NF-κB signaling can be detected in as much as 95% of all cancer cases and is responsible for aberrant expression of huge battery of genes involved in cancer onset and progression [17]. Natural compounds have been shown to counteract with NF-κB signaling and down-regulate its activity, thus acting as potent agents in both, cancer prevention and treatment [3,19,20,21,22,23].

A transannular sulfur bridge as present in compound **1** seems to be essential for the observed NF-κB pathway interfering activity, as the related compound **2** without this feature did not display any activity. The finding of 6-acetylmonodethiogliotoxin with a monosulfide bridge is of importance, as this compound may serve as lead for the development of more potent and selective ligands for this therapeutically interesting drug target.

It is interesting to note, that monosulfides of gliotoxin derivatives have been obtained semi-synthetically from disulfid gliotoxin derivatives by reaction with tervalent phosphorus compounds, e.g., triphenylphosphine [24]. The investigated marine derived strain of *Dichotomomyces cejpii* however constantly produced 6-acetylmonodethiogliotoxin (**1**) under different cultivation conditions that was isolated directly from the fungal extract as a natural product. To our knowledge there are only sirodesmin H, isolated from the fungus *Phoma lingam* and emestrin-G, isolated from the fungus *Armillaria tabescens*, respectively, where such a bridged monosulfid was obtained directly by isolation as a natural product [25,26]. However these compounds solely possess the same epimonothiodioxopiperazine moiety while the rest of the structural backbone is quite different.

## 3. Experimental Section

### 3.1. General Procedures for Natural Products Chemistry

Optical rotations were measured with a Jasco DIP 140 polarimeter (Jasco Inc., Easton, CA, USA). UV and IR spectra were obtained employing Perkin-Elmer Lambda 40 (PerkinElmer, Inc., Waltham, MA, USA) and Perkin-Elmer Spectrum BX instruments (PerkinElmer, Inc., Waltham, MA, USA), respectively. ^1^H and ^13^C NMR spectra were recorded on a Bruker Avance 300 DPX spectrometer (Bruker Corporation, Billerica, MA, USA). NMR spectra were referenced to residual solvent signals of acetone and methanol at δ_H/C_ 2.04/29.8 and 3.35/49.0, respectively. VLC grade (40–63 µm) Si gel (FLUKA, St. Louis, MO, USA) was used for vacuum liquid chromatography. All organic solvents were distilled prior to use. Low-Resolution Electrospray Ionisation Mass Spectrometry (LRESIMS) measurements were performed employing an API 2000, Triple Quadrupole LC/MS/MS (Applied Biosystems, Saint Aubin, Essone, France /MDS Sciex, Darmstadt, Germany) with Electrospray Ionisation (ESI) source. HRESIMS were recorded on a Bruker Daltonik micrOTOF-Q time-of-flight mass spectrometer with ESI source (Bruker Corporation, Billerica, MA, USA). High Performance Liquid Chromatography (HPLC) was carried out using different HPLC systems: A: Waters system, controlled by Waters Millenium software (Milford, MA, USA), consisting of a 600E pump (Waters Corp., Milford, MA, USA), a 996 PDA (Waters Corp., Milford, MA, USA), and a 717 plus autosampler (Waters Corp., Milford, MA, USA); B: HPLC system controlled by Merck Hitachi Model D-7000 Chromatography Data Station Software HPLC System Manager Version 4.0 software (Merck KGaA, Darmstadt Germany), consisting of L6200 A intelligent pump (Merck KGaA, Darmstadt, Germany), D 6000 interface (Merck KGaA, Darmstadt, Germany) and an L4500 PDA (Merck KGaA, Darmstadt, Germany). C: controlled by HP ChemStation for LC Rev.A.06.03[909] software (Hewlett-Packard Development Company, Palo Alto, CA, USA), consisting of a L-7100 Merck Hitachi pump (Merck KGaA, Darmstadt, Germany) and a HP-series 1050 detector (Hewlett-Packard Development Company, L.P., Palo Alto, CA, USA). D: HPLC composed of a Waters 515 pump (Waters Corp., Milford, MA, USA) together with a Knauer K-2300 differential refractometer (KNAUER Wissenschaftliche Geräte GmbH, Berlin, Germany).

### 3.2. Cultivation of Fungus, Extraction and Isolation

The marine-derived fungus *Dichotomomyces cejpeii* was isolated from a sample of the sponge *Callyspongia* sp. *cf. C. flammea* (collected at Bare Island, Sydney, Australia). The isolation of the fungus was carried out using an indirect isolation method. Sponge samples were rinsed three times with sterile H_2_O. After surface sterilization with 70% EtOH for 15 s the sponge was rinsed in sterile artificial seawater (ASW). Subsequently, the sponge material was aseptically cut into small pieces and placed on agar plates containing isolation medium: agar 15 g/L, ASW 800 mL/L, glucose 1 g/L, peptone from soymeal 0.5 g/L, yeast extract 0.1 g/L, benzylpenicillin 250 mg/L, and streptomycin sulfate 250 mg/L. The fungus growing out of the spongeal tissue was separated on biomalt medium (biomalt 20 g/L, agar 10 g/L, ASW 800 mL/L) until the culture was pure. The isolated fungus was identified by P. Massart and C. Decock, BCCM/MUCL, Catholic University of Louvain, Louvain-la-Neuve, Belgium. A specimen is deposited at the Institute for Pharmaceutical Biology, University of Bonn, isolation number “293K09”, strain number 225.

The fungus was cultivated on cultivation medium MPY: 10 L of malt, peptone, yeast solid agar medium (malt extract 20 g/L, peptone 2.5 g/L, yeast extract 2.5 g/L and 15 g/L agar) supplemented with demineralized water in 40 Fernbach flasks at room temperature (RT) for 30 days.

Fungal biomass and media were homogenized using an Ultra-Turrax apparatus (IKA Werke GmbH & Co. KG, Baden-Württemberg, Germany) and extracted with 5 L ethyl acetate (EtOAc) to yield 4.1 g of crude extract. This material was fractionated by Normal Phase (NP) vacuum liquid chromatography (VLC) using a stepwise gradient solvent system of increasing polarity starting with 100% CH_2_Cl_2_ to 100% EtOAc and to 100% methanol (MeOH) which yielded 10 fractions. Fraction 2 was separated via NP VLC, using stepwise elution with CH_2_Cl_2_–petroleum ether (50–50) to 100% CH_2_Cl_2_, and 100% MeOH which yielded 15 fractions. VLC fraction 1 contained compounds **1**–**3** and was distributed between hexane (100%) and MeOH–H_2_O (60–40) using a solvent/solvent extraction three times. The methanolic phase was further separated using a Reversed Phase C18 (RP_18_) VLC column and eluted stepwise with H_2_O–MeOH (80–20) to 100% MeOH, 100% DCM which yielded 8 fractions. Subfraction 1.3 was further separated with a second NP VLC column, eluted stepwise with petroleum ether–acetone (95–5), to 100% acetone, and acetone–MeOH (50–50) which yielded 8 fractions. Subfraction 1.3.3 contained compounds **1**–**3**. Compound **1** (6-acetylmonodethiogliotoxin) was isolated from subfraction 1.3.3 via NP-HPLC system D (Knauer Eurosphere II Si column, 250 mm × 4.6 mm) (Knauer Wissenschaftliche Geräte GmbH, Berlin, Germany) with petroleum ether–acetone (85–15), 1.0 mL/min, as a yellowish white powder, *t*_R_: 22 min in to batches with a total amount of 19.5 mg.

Compound **2** was found in VLC fraction 4 and subfraction 1.3.3. Subfraction 1.3.3 was separated as described above and compound **2** (6-acetylbisdethiobis(methylthio)gliotoxin) was obtained as yellowish white powder, *t*_R_: 30.2 min (98.9 mg). Minor amounts were isolated from VLC fraction 4 via RP_8_-HPLC system D (Knauer Eurosphere II RP_8_ column, 250 mm × 8 mm) (Knauer Wissenschaftliche Geräte GmbH, Berlin, Germany) with H_2_O–MeOH (50–50), 1.5 mL/min, *t*_R_: 15.5 min (4.5 mg).

Compound **3** was also obtained from subfraction 1.3.3, which was in a first round separated as described above. From this separation, subfraction 1.3.3.3 (*t*_R_:18.2 min) resulted, and was further purified using NP-HPLC system D (Knauer Eurosphere II Si column, 250 mm × 4.6 mm) (KNAUER Wissenschaftliche Geräte GmbH, Berlin, Germany) with petroleum ether–acetone (90–10), 1.0 mL/min, and compound **3** (5a,6-anhydrobisdethiobis(methylthio)gliotoxin) was isolated as yellowish white powder, *t*_R_: 33.5 min (4.6 mg).

Compound **4** was found in subfractions 1.3.1, 1.3.2 and 1.3.3. Subfraction 1.3.1 was separated with NP-HPLC system D (Knauer Eurosphere Si, 250 mm × 8 mm) (KNAUER Wissenschaftliche Geräte GmbH, Berlin, Germany) using petroleum ether–acetone (90–10), flow 1.0 mL/min. The resulting subfraction 1.3.1.3 (*t*_R_: 8.0 min) was further purified via RP_18_-HPLC system B with (Macherey-Nagel Nucleodur RP_18_ column, 250 mm × 4.6 mm) (Macherey-Nagel GmbH & Co. KG, North-Rhine-Westphalia, Germany) H_2_O–ACN (43–57), flow: 1.7 mL/min, *t*_R_: 8.0 min to obtain heveadride (**4**) (30.0 mg). Subfraction 1.3.2 was purified via NP-HPLC system D (Knauer Eurosphere Si column, 250 mm × 8 mm) (Knauer Wissenschaftliche Geräte GmbH, Berlin, Germany) with petroleum ether–acetone (90–10), flow 1.0 mL/min, *t*_R_: 8.0 min, and heveadride (**4**) was isolated as white powder (35.0 mg). Subfraction 1.3.3 was separated via NP-HPLC system D (Knauer Eurosphere II Si column, 250 mm × 4.6 mm) (Knauer Wissenschaftliche Geräte GmbH, Berlin, Germany) with petroleum ether–acetone (85–15), 1.0 mL/min, and heveadride (**4**) was isolated as white powder, *t*_R_: 6.0 min (12.9 mg).

**6-Acetylmonodethiogliotoxin** (**1**): yellowish white amorphous compound (19.5 mg; 2.0 mg/L); ${\left[\alpha \right]}_{D}^{25}$ −4.9 (*c* 0.34, MeOH); UV (MeOH ), λ_max_ (log ε) 203 (3.62), 262 (3.28) nm; IR (ATR) *ν_max_* 3425, 2922, 1722, 1368, 1234, 1047, 877, 719, 668, 628 cm^−1^; ^1^H and ^13^C NMR data (see Table 1); ESI-MS *m*/*z* 337 [M +H ]^+^, 354 [M + NH_4_]^+^; HRESI-MS *m*/*z* 359.0672 [M + Na]^+^ (calcd. for C_15_H_16_N_2_O_5_SNa, *m*/*z* 359.0678).

**6-Acetylbisdethiobis(methylthio)gliotoxin** (**2**): yellowish white amorphous compound (94.4 mg; 9.4 mg/L); ${\left[\alpha \right]}_{D}^{25}$ −62.9 (*c* 0.28, MeOH); UV (MeOH), λ_max_ (log ε) 203 (3.95), 269 nm (3.42) nm; IR (ATR) ν_max_ 3425, 2922, 1734, 1648, 1418, 1386, 1237, 1189, 1142, 1051, 979, 878, 668, 600, 534 cm^−1^; ^1^H and ^13^C NMR data (see Table 1); ESI-MS *m*/*z* 416 [M + NH_4_]^+^, 421 [M + Na]^+^, 819 [2M + Na]^+^; HRESI-MS *m*/*z* 421.0801 [M + Na]^+^ (calcd. for C_17_H_22_N_2_O_5_S_2_Na, 421.0868).

**5a,6-Anhydrobisdethiobis(methylthio)gliotoxin** (**3**): yellowish white amorphous compound (4.6 mg; 0.5 mg/L). ${\left[\alpha \right]}_{D}^{25}$ −65.1 (*c* 0.36, MeOH); ^1^H and ^13^C NMR data (see Table 1); ESI-MS *m*/*z* 361 [M + Na]^+^, 700 [2M + Na]^+^; HRESI-MS *m*/*z* 361.0662 [M + Na]^+^ (calcd. for C_15_H_18_N_2_O_3_S_2_Na, 361.0651).

**Heveadride** (**4**): white crystalline compound (77.9 mg; 7.8 mg/L). ${\left[\alpha \right]}_{D}^{25}$ +20.9 (*c* 2.22, MeOH) [lit value ${\left[\alpha \right]}_{D}^{25}$ +63 (*c* 1.19, CH_2_Cl_2_) [27]]; ^1^H and ^13^C NMR data (see Table S3); ESI-MS *m*/*z* 333 [M + H]^+^, 331 [M − H]^−^.

### 3.3. Bioassays

#### 3.3.1. Cell Culture

K562 (human chronic myeloid leukemia) and Jurkat (T-cell leukemia) cells (DSMZ) were cultured in RPMI 1640 medium (Lonza, Verviers, Belgium) supplemented with 10% (v/v) fetal calf serum (Lonza, Verviers, Belgium) and 1% (v/v) antibiotic-antimycotic (Bio-Whittaker, Lonza, Verviers, Belgium) at 37 °C and 5% CO_2_, humidified atmosphere. Cells were harvested every 3 days.

#### 3.3.2. Transient Transfection and Luciferase Reporter Gene Assay

K562 cells were transiently transfected as described previously [28]. For each electroporation, we used 5 µg of a luciferase reporter gene construct containing 5 repeats of a consensus NF-κB site (Stratagene, Genomics Agilent, Diegem, Belgium) and 5 µg of a *Renilla* luciferase plasmid (Promega, Leiden, The Netherlands). The ICAM-1 LUC reporter plasmid was a generous gift from Wim Vanden Berghe (University of Antwerp, Antwerp, Belgium). The full-length ICAM-1 promoter construct contains approximately 1.4 kb of ICAM-1 5′-flanking DNA linked to the firefly luciferase (LUC) gene. Promoter sequences between 393 and 176 bp upstream of the gene, containing binding sites for C/EBP and NF-κB. After electroporation, cells were re-suspended in RPMI 1640 culture medium (Lonza, Verviers, Belgium), 10% FCS, 1% AB and cultured at 37 °C and 5% CO_2_ for 24 h. Afterwards, cells were harvested and re-suspended in fresh growth medium (RPMI 1640, 0.1% FCS, 1% AB) to a final concentration of 1 × 10^6^ cells/mL and pre-treated for 2 h with 6-acetylmonodethiogliotoxin at indicated concentrations, followed by TNFα activation (20 ng/mL) for 6 h. After incubation, 75 µL of Dual-Glo^™^ Luciferase Reagent (Promega, Leiden, The Netherlands) was added to 75 µL of the cellular suspension for a 10 min at 22 °C before luciferase activity measurement. Subsequently, 75 μL of Dual-Glo^™^ Stop&Glo^®^ Reagent (Promega, Leiden, The Netherlands) was added for 10 min at 22 °C to the cell suspension to measure *Renilla* activity. An Orion microplate luminometer (Berthold Technologies, Bad Wildbad, Germany) was used to measure luciferase and *Renilla* activity. The results are expressed as a ratio of arbitrary units of firefly luciferase activity to *Renilla* luciferase activity.

#### 3.3.3. Cell Viability Assessment

To assess percentage of viable K5562 cells within sample and to determinate K562 cells proliferation trypan blue exclusion test was used. Trypan blue is a vital stain that belongs to the family of azo compounds. It is a selective dye that stains only dead cells, passing through their plasma membrane. Viable cells are unstained as they can actively extrude this dye. Briefly, 20 μL of cell suspension was mixed with 20 μL of trypan blue solution and evaluated by Malassez cell counting chamber (Fisher Scientific, Erembodegem, Belgium). In order to assess the cell viability, the percentage of unstained cells to the total amount of cells within the sample was calculated and normalized to 100% of control cells viability. In order to assess cell proliferation the concentration of unstained cells was determined and normalized to 100% of control cells concentration.

#### 3.3.4. Extraction of Cellular Proteins

After the indicated incubation times with 6-acetylmonodethiogliotoxin and TNFα, Jurkat cells were lysed, and the nuclear and cytoplasmic extracts were prepared according to Duvoix *et al.* [28]. Briefly, cell pellets (10^7^ cells per sample) were suspended in ice-cold hypotonic lysis buffer containing protease inhibitor cocktail (Complete^®^, Roche, Prophac, Luxembourg) and incubated on ice for 15 min. After incubation, 10% Igepal (Sigma-Aldrich BVBA Diegem, Belgium) was added to the cell suspension and each microcentrifuge tube was vigorously mixed by Vortex for 10 s to lyse cells followed by centrifugation in a refrigerated microcentrifuge tube at 18,000 rcf for 1 min. The cytoplasmic extract (supernatant) was aliquoted and stored at −80 °C until use. Cell pellets were additionally washed with 100 μL of hypotonic lysis buffer and after centrifugation at 4 °C, 18,000 rcf for 2 min the supernatant was removed. The ice-cold nuclear extraction buffer was added to each pellet and the cell suspension was gently mixed on an orbital shaker for 15 min at 4 °C following centrifugation at 10,500 rcf for 7 min at 4 °C. Then nuclear extracts (supernatant) were transferred to pre-chilled microcentrifuge tubes and stored at −80 °C until use. Protein content was determined for each sample using the Bradford assay (Bio-Rad protein Assay, Biorad, Nazareth, Belgium).

#### 3.3.5. Western Blot Analysis

Proteins of nuclear and cytoplasmic extracts were separated by size using sodium dodecyl sulfate polyacrylamide gel electrophoresis (SDS-PAGE, 10%), transferred onto nitrocellulose membranes and blocked with 5% non-fat milk in phosphate buffered saline (PBS)-Tween overnight. Blots were then incubated with primary antibodies: anti-IκBα (1/500 Santa Cruz SC-371, Tebu-Bio, Boechout, Belgium), anti-p50 (1/5000, Santa Cruz SC-7178X), anti-p65 (1/5000, Santa Cruz SC-8008), anti-α-tubulin (1/5000, Calbiochem CP06, VWR, Leuven, Belgium) or anti-lamin B (1/1000, Santa Cruz SC-6216). All antibodies were diluted in a PBS-Tween solution containing 5% bovine serum albumin (BSA) or 5% milk according to the providers’ protocols. After incubation with primary antibodies, membranes were washed 3 × 10 min with PBS-Tween followed by an incubation of 1 h at RT with the corresponding secondary (HRP-conjugated) antibodies. After washing 3 × 10 min with PBS-Tween, specific immunoreactive proteins were visualized by autoradiography using the ECL Plus Western Blotting Detection System Kit^®^ (GE Healthcare, Roosendaal, The Netherlands). Lamin B for cytoplasmic extracts and α-tubulin for nuclear extracts, were used as loading controls.

#### 3.3.6. TransAM Assay

Jurkat cells were seeded at concentration 3 × 10^5^ cells/mL and 10^7^ cells were treated with Alterporriol E for 4 h followed by activation with TNFα (20 ng/mL) for 30 min. Subsequently, nuclear proteins were extracted from the cells according to manufacturer’s protocol (Active motif, Nuclear extract kit, La Hulpe, Belgium). Nuclear protein extracts were submitted for TransAM assay, which was conducted according to the manufacturer’ instruction (Active motif, TransAM NFκB family, La Hulpe, Belgium) and luminescent signal was measured by Luminometer (CentroLB 960, Berthold Technologies, Bad Wildbad, Germany).

#### 3.3.7. Statistical Analysis

Data are expressed as mean ± SD Significance was determined by the Student’s *t*-tests. *p*-Values below 0.05 were considered as statistically significant. IC_50_ values were calculated using XY scatter dependency chart. The 50% inhibition activity on NF-κB expression was established by using the best fitting model and calculated by trend formulas. The average value of at least 3 independent experiments was applied. Data are expressed as mean ± SD Significance was determined by the Student’s *t*-tests. *p*-Values below 0.05 were considered as statistically significant.

## 4. Conclusions

Overall the described monosulfid production seems to be a special feature of this particular marine derived strain of *D. cejpii.* Moreover, our study validates the inhibitory effects of newly isolated natural epipolythiodiketopiperazines on anti-proliferative mechanisms *via* inhibition of TNFα-induced NF-κB activity. Especially, 6-acetylmonodethiogliotoxin as a most potent NF-κB inhibitor presenting the strongest anti-proliferative effects within the evaluated compounds represents a promising future marine-derived cytostatic drug candidate.

## Supplementary Files

Supplementary File 1
