# Supplementary material for: Epipolythiodiketopiperazines from the Marine Derived Fungus Dichotomomyces cejpii with NF-κB Inhibitory Potential
Source: Mar Drugs. 2015 Aug 6;13(8):4949–66. doi: 10.3390/md13084949 (PMC4557009; doi:10.3390/md13084949)
Supplement: Supplementary File 1 [file marinedrugs-13-04949-s001.doc]

**Supplementary Information**

**Contents**

| **Screened cultivation media** | S2 |
| --- | --- |
| **Compound characterization:** | S3 |
| **Figure S1.1.** UV spectrum of compound **1** in MeOH | S3 |
| **Figure S1.2.** IR spectrum of compound **1** | S3 |
| **Figure S1.3.** 1H NMR spectrum of compound **1** | S4 |
| **Figure S1.4.** 1H NMR spectrum of compound **1** after reisolation | S4 |
| **Figure S1.5.** 13C NMR spectrum of compound **1** | S4 |
| **Figure S1.6.** Dept 135 NMR spectrum of compound **1** | S5 |
| **Figure S1.7.** 1H-13C HSQC 2D-NMR spectrum of compound **1** | S5 |
| **Figure S1.8.** 1H-1H COSY 2D-NMR spectrum of compound **1** | S6 |
| **Figure S1.9.** 1H-13C HMBC 2D-NMR spectrum of compound **1** | S6 |
| **Figure S1.10.** 1H-1H NOESY 2D-NMR spectrum of compound **1** | S7 |
| **Figure S1.11.** HRESIMS spectrum for compound **1** | S7 |
| **Figure S2.1.** UV spectrum of compound **2** | S8 |
| **Figure S2.2.** IR spectrum of compound **2** | S8 |
| **Figure S2.3.** 1H NMR spectrum of compound **2** | S9 |
| **Figure S2.4.** 13C NMR spectrum of compound **2** | S9 |
| **Figure S2.5.** Dept 135 NMR spectrum of compound **2** | S9 |
| **Figure S2.6.** 1H-13C HSQC 2D-NMR spectrum of compound **2** | S10 |
| **Figure S2.7.** 1H-1H COSY 2D-NMR spectrum of compound **2** | S10 |
| **Figure S2.8.** 1H-13C HMBC 2D-NMR spectrum of compound **2** | S11 |
| **Figure S2.9.** 1H-1H NOESY 2D-NMR spectrum of compound **2** | S11 |
| **Figure S2.10.** HRESIMS spectrum for compound **2** | S12 |
| **Figure S3.1.** 1H NMR spectrum of compound **3** | S13 |
| **Figure S3.2.** 13C NMR spectrum of compound **3** | S13 |
| **Figure S3.3.** Dept 135 NMR spectrum of compound **3** | S13 |
| **Figure S3.4.** 1H-13C HSQC 2D-NMR spectrum of compound **3** | S14 |
| **Figure S3.5.** 1H-1H COSY D-NMR spectrum of compound **3** | S14 |
| **Figure S3.6.** 1H-13C HMBC 2D-NMR spectrum of compound **3** | S15 |
| **Figure S3.7.** 1H-1H NOESY 2D-NMR spectrum of compound **3** | S15 |
| **Figure S3.8.** HRESIMS spectrum for compound **3** | S16 |
| **Tables with Spectroscopic Data** | S16 |
| **Table S1.** NMR Spectroscopic Data for compound **1** | S16 |
| **Table S2.** NMR Spectroscopic Data for compound **2** | S17 |
| **Table S3.** NMR Spectroscopic Data for compound **3** | S17 |
| **Purity data** | S18 |
| **Purity data for compound 1:** | S18 |
| **Table S4.** Purity analysis of compound **1** with HPLC | S18 |
| **Figure S4.1.** HPLC chromatogram of compound **1** method A (265 nm) | S18 |
| **Figure S4.2.** HPLC chromatogram of compound **1** method B (280 nm) | S19 |
| **Figure S4.3.** HPLC chromatogram of compound **1** method B (250 nm) | S19 |
| **Figure S4.4.** HPLC chromatogram of compound **1** method B (265 nm) | S20 |
| **Purity data for compound 1:** | S20 |
| **Table S5.** Purity analysis of compound **1** with HPLC after reisolation | S20 |
| **Figure S4.5.** HPLC chromatogram of compound **1** method C after reisolation (265 nm) | S20 |
| **Figure S4.6.** HPLC chromatogram of compound **1** method B (240 nm) | S21 |
| **Purity data of compound 2:** | S21 |
| **Table S6.** Purity analysis of compound **2** with HPLC | S21 |
| **Figure S5.1.** HPLC chromatogram of compound **2** method A (250 nm) | S22 |
| **Figure S5.2.** HPLC chromatogram of compound **2** method A (280 nm) | S22 |
| **Figure S5.3.** HPLC chromatogram of compound **2** method B (250 nm) | S23 |
| **Figure S5.4.** HPLC chromatogram of compound **2** method B (280 nm) | S23 |
| **Purity data for compound 4:** | S24 |
| **Table S7.** Purity analysis of compound **4** with HPLC | S24 |
| **Figure S6.1.** HPLC chromatogram of compound **4** method A (240 nm) | S24 |
| **Figure S6.2.** HPLC chromatogram of compound **4** method A (270 nm) | S24 |
| **Figure S6.3.** HPLC chromatogram of compound **4** method A (240 nm) | S25 |
| **Figure S6.4.** HPLC chromatogram of compound **4** method A (270 nm) | S25 |
| **NMR Key correlations** | S25 |
| **Figure S7.1.** NMR-Key correlations in compound **1** | S25 |
| **Figure S7.2.** NOESY-Key correlations in compound **1** | S26 |
| **Figure S7.3.** NMR-Key correlations in compound **2** | S26 |
| **Figure S7.4.** NOESY-Key correlations in compound **2** | S26 |
| **Figure S7.5.** NMR-Key correlations in compound **3** | S27 |
| **Figure S7.6.** NOESY-Key correlations in compound **3** | S27 |

Screened Cultivation Media

- **BMS** medium: 15 g/L agar, 20 g/L biomalt extract, ad 1 L ASW (artificial sea water: 0.1 g/L KBr, 23.48 g/L NaCl, 10.61 g/L MgCl_2_·6H_2_O, 1.47 g/L CaCl_2_·2H_2_O, 0.66 g/L KCl,
  0.04 g/L SrCl_2_·6H_2_O, 3.92 g/L Na_2_SO_4_, 0.19 g/L NaHCO_3_, and 0.1 g/L H_3_BO_3_).
- **MPY** medium: 15 g/L agar, malt extract 20 g/L, peptone 2.5 g/L, yeast extract
  2.5 g/L) ad 1 L demineralized water.
- **REA** medium (without polysorbate 80): 20 g/L agar 20 g/L rice for rice extract, ad 1 L demineralized water.
- **Tennelin** medium: agar 15 g/L, mannitol 50 g/L, 5 g KNO_3_, 1g KH_2_PO_4_, 0.5g MgSO_4_·7H_2_O, 0.1 g NaCl, 0.2 g CaCl_2_, 20 mg FeSO_4_·7H_2_O, 10 mL Liquid 2: [ZnSO_4_·7H_2_O 880 mg/L, CuSO_4_·5H_2_O, 40 mg/L MnSO_4_·4H_2_O, 7.5 mg/L, Boracid, 6 mg/L, 4 mg/L (NH_4_)_6_Mo_7_O_4_·H_2_O ad 1 L Aqua dem.], ad 1 L demineralized water.

Compound Characterization:


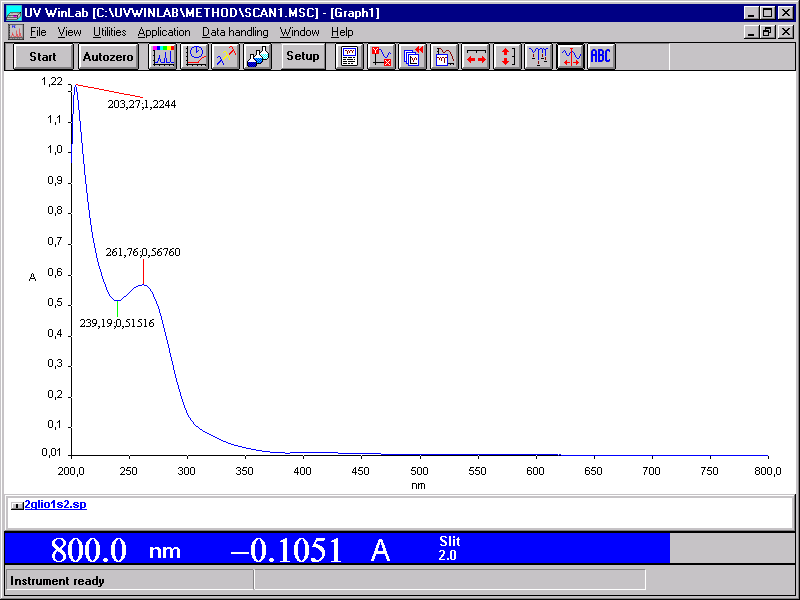


**Figure S1.1.** UV spectrum of compound **1**.

**
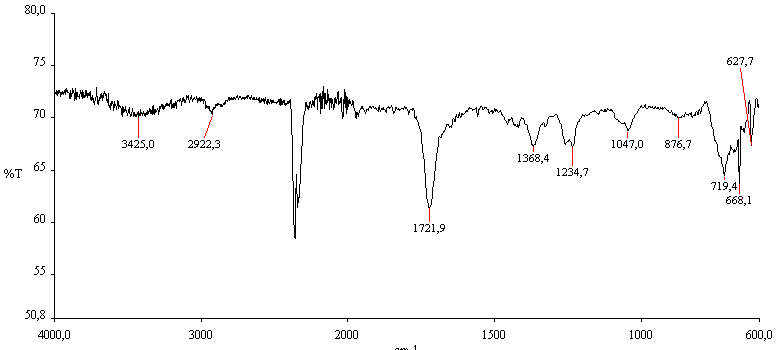
**

**Figure S1.2.** IR spectrum of compound **1**.

*
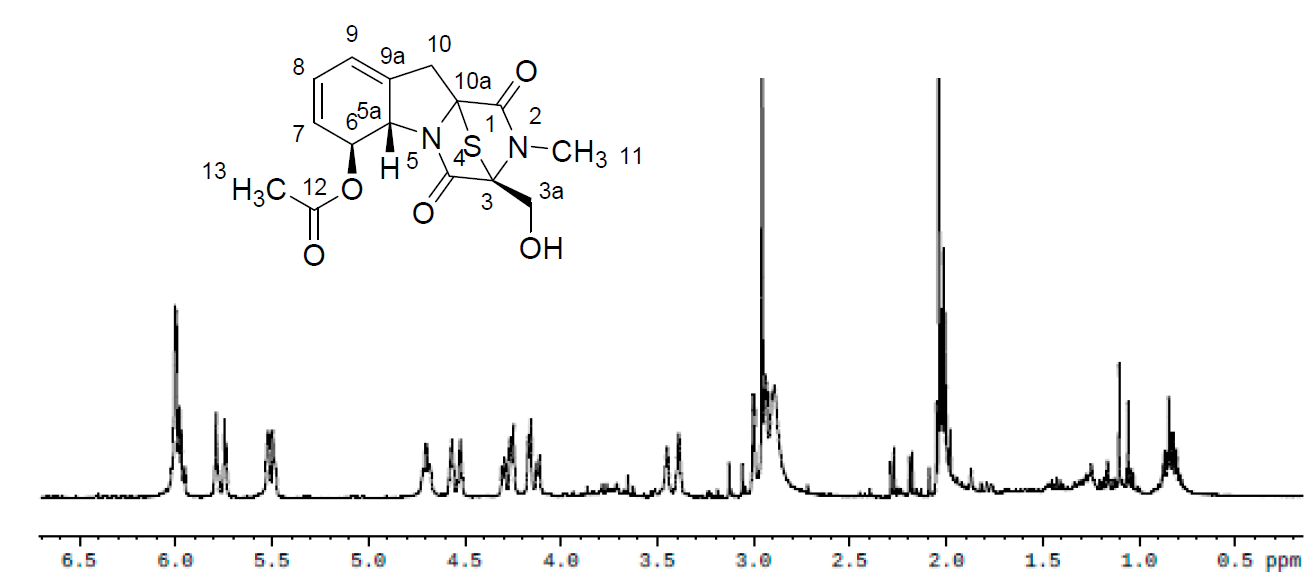
*

**Figure S1.3.** ^1^H NMR spectrum of compound **1** in acetone-*d*_6_.


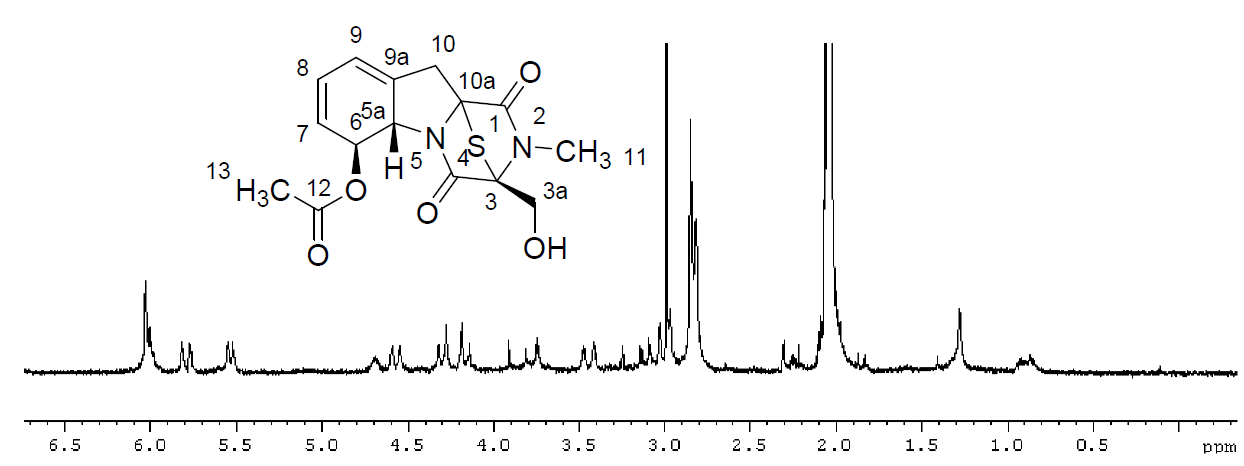


**Figure S1.4.** ^1^H NMR spectrum of compound **1** in acetone-*d_6_* after reisolation.


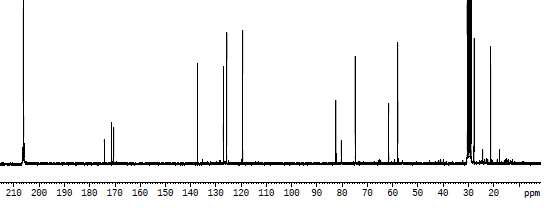


**Figure S1.5.** ^13^C NMR spectrum of compound **1** in acetone-*d*_6_.


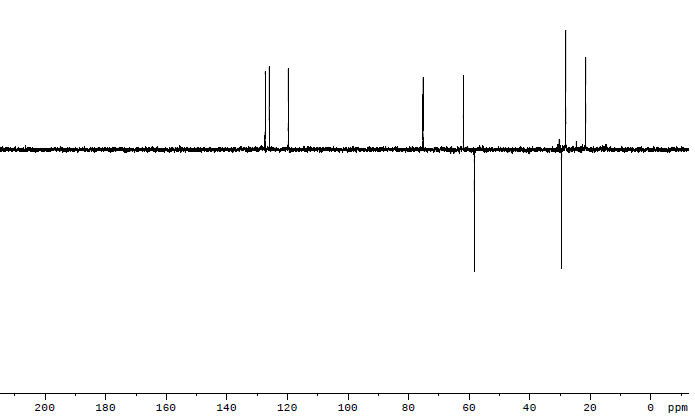


**Figure S1.6.** Dept 135 NMR spectrum of compound **1** in acetone-*d*_6_.


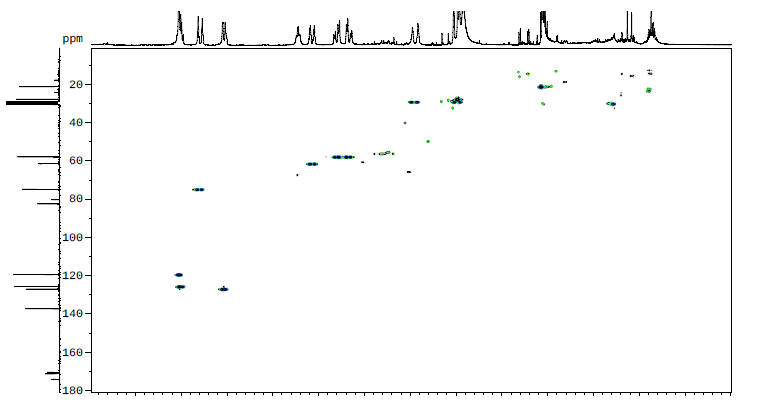


**Figure S1.7.** ^1^H-^13^C HSQC 2D-NMR spectrum of compound **1** in acetone-*d*_6_.


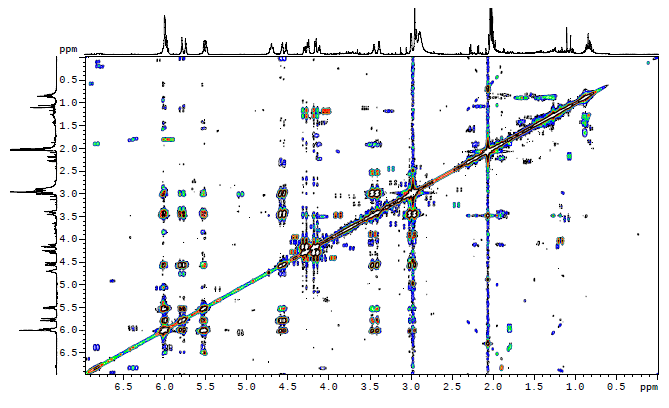


**Figure S1.8.** ^1^H-^1^H COSY 2D-NMR spectrum of compound **1** in acetone-*d*_6_.


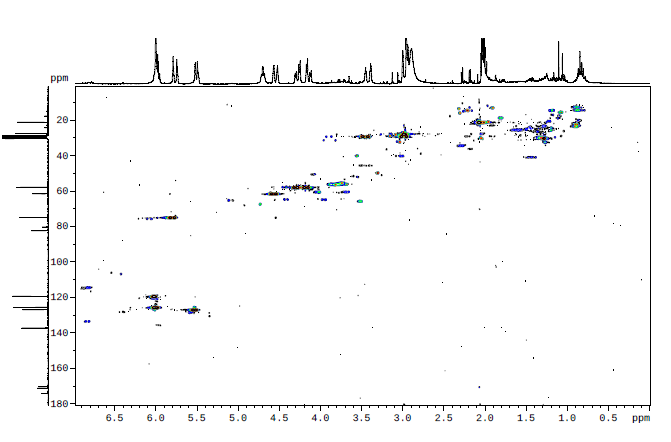


**Figure S1.9.** ^1^H-^13^C HMBC 2D-NMR spectrum of compound **1** in acetone-*d_6_*.


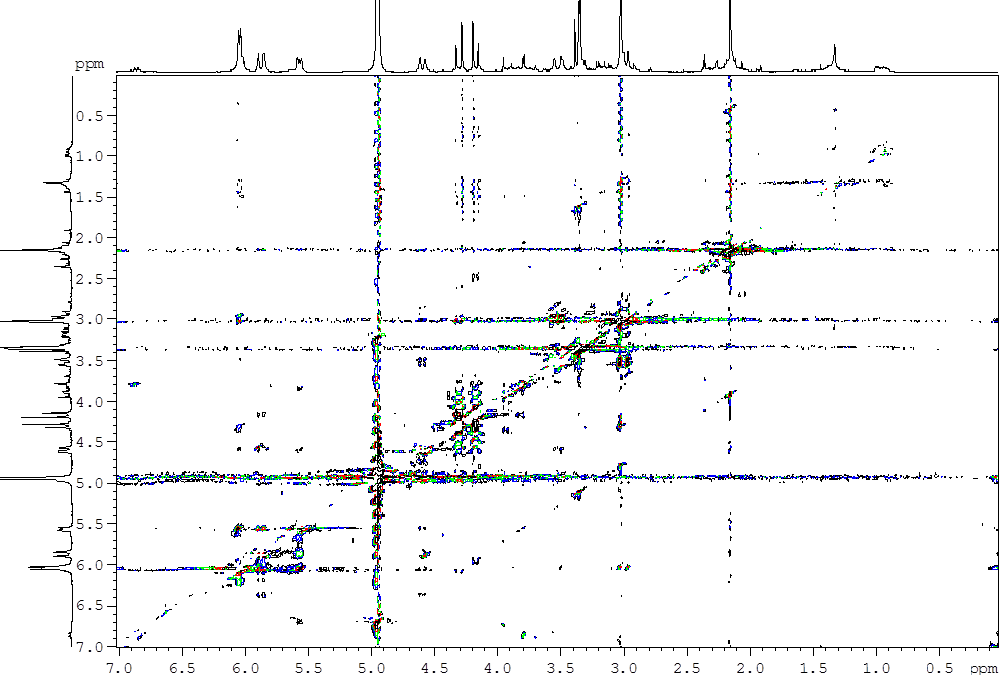


**Figure S1.10.** ^1^H-^1^H NOESY 2D-NMR spectrum of compound **1** in methanol-*d*_4_.

**Figure S1.11.** HRESIMS spectrum for compound **1**.


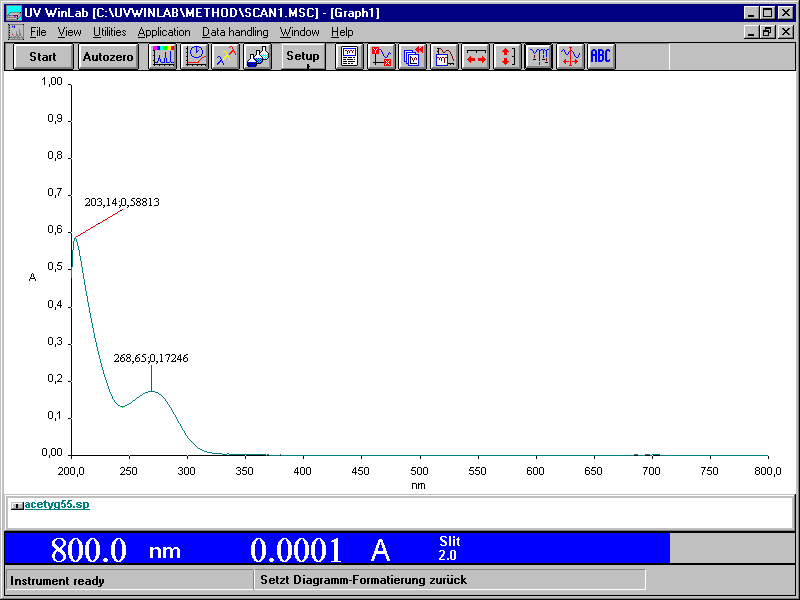


**Figure S2.1.** UV spectrum of compound **2** in MeOH.


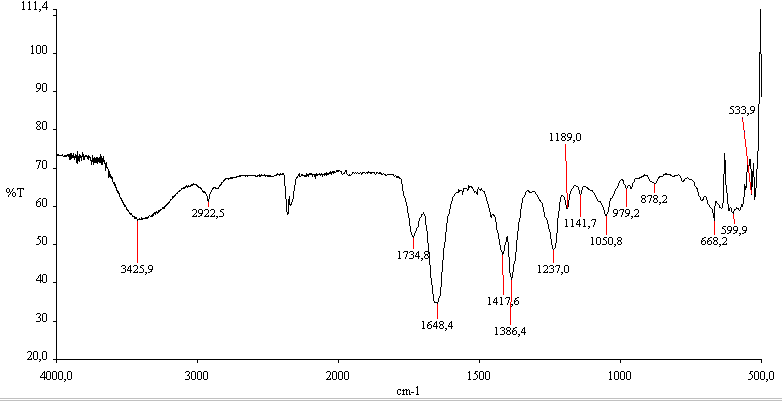


**Figure S2.2.** IR spectrum of compound **2**.


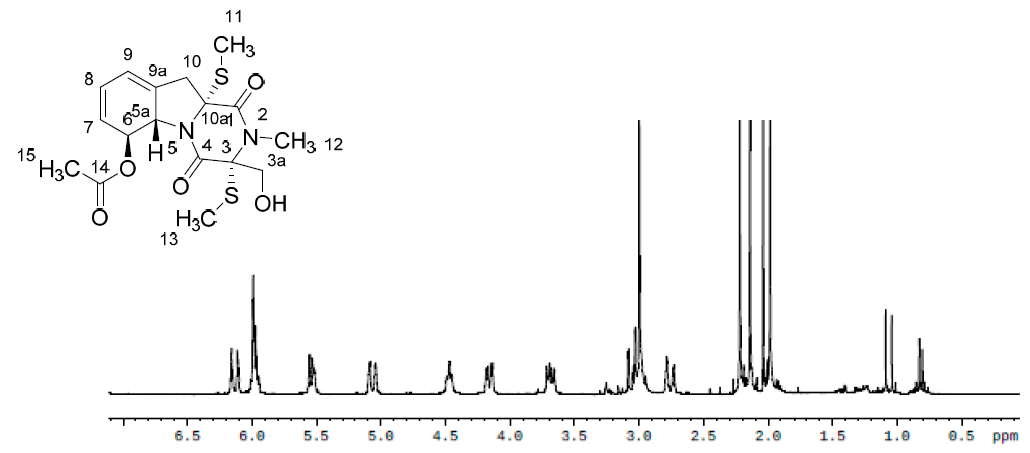


**Figure S2.3.** ^1^H NMR spectrum of compound **2** in acetone-*d_6_*.


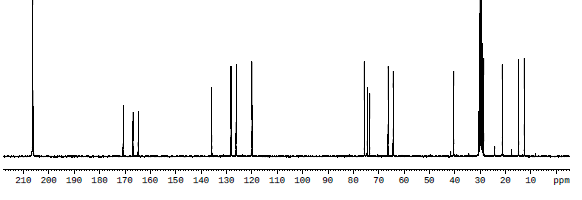


**Figure S2.4.** ^13^C NMR spectrum of compound **2** in acetone-*d*_6_.


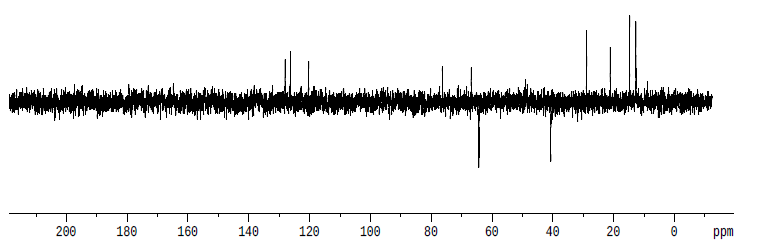


**Figure S2.5.** Dept 135 NMR spectrum of compound **2** in acetone-*d*_6_.

**
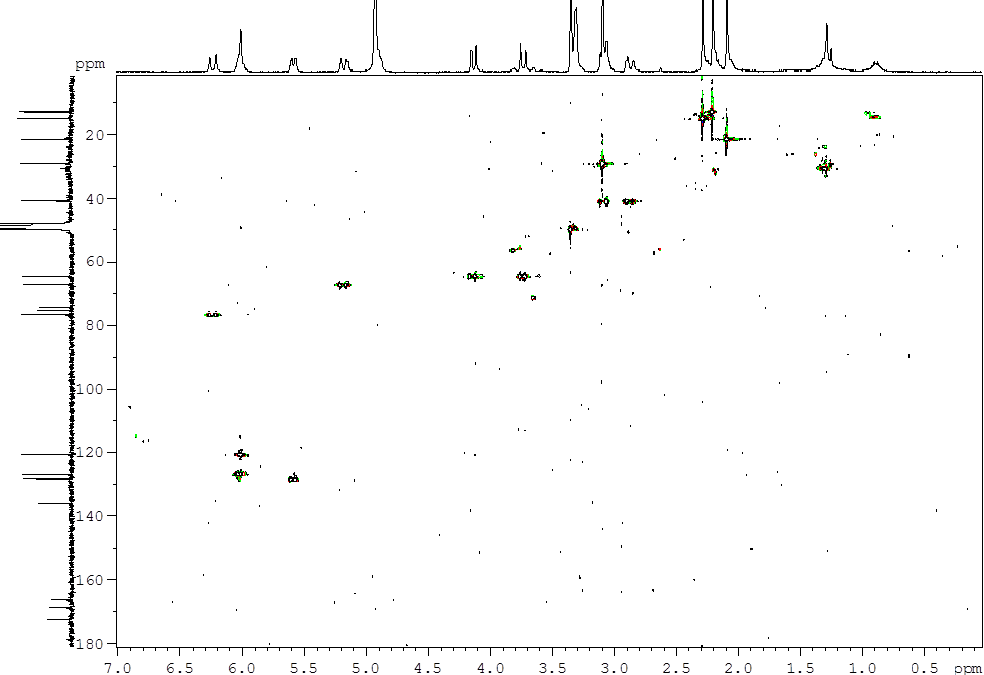
**

**Figure S2.6.** ^1^H-^13^C HSQC 2D-NMR spectrum of compound **2** in methanol-*d*_4_.

**
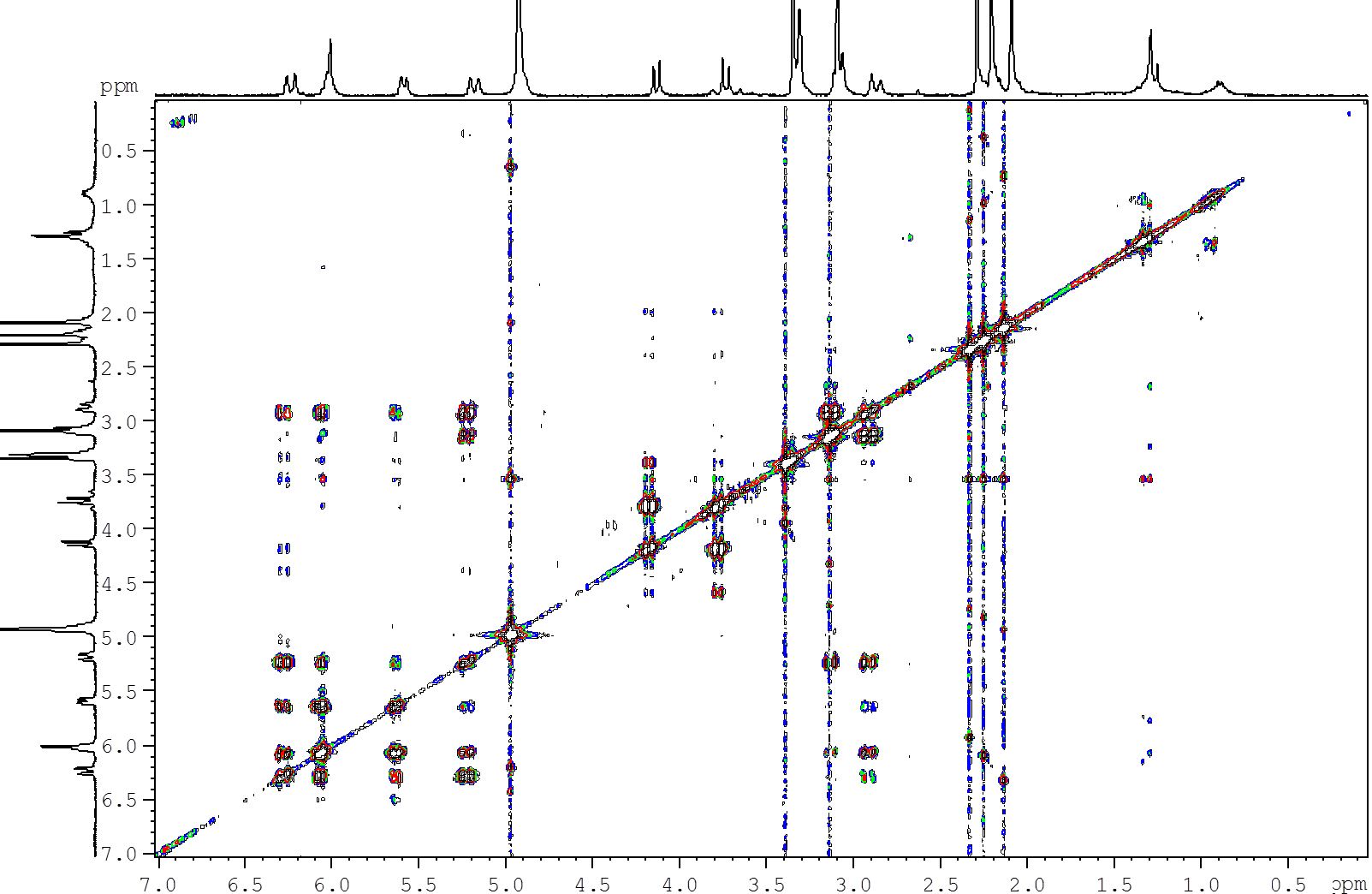
**

**Figure S2.7.** ^1^H-^1^H COSY 2D-NMR spectrum of compound **2** in methanol-*d*_4_.

**
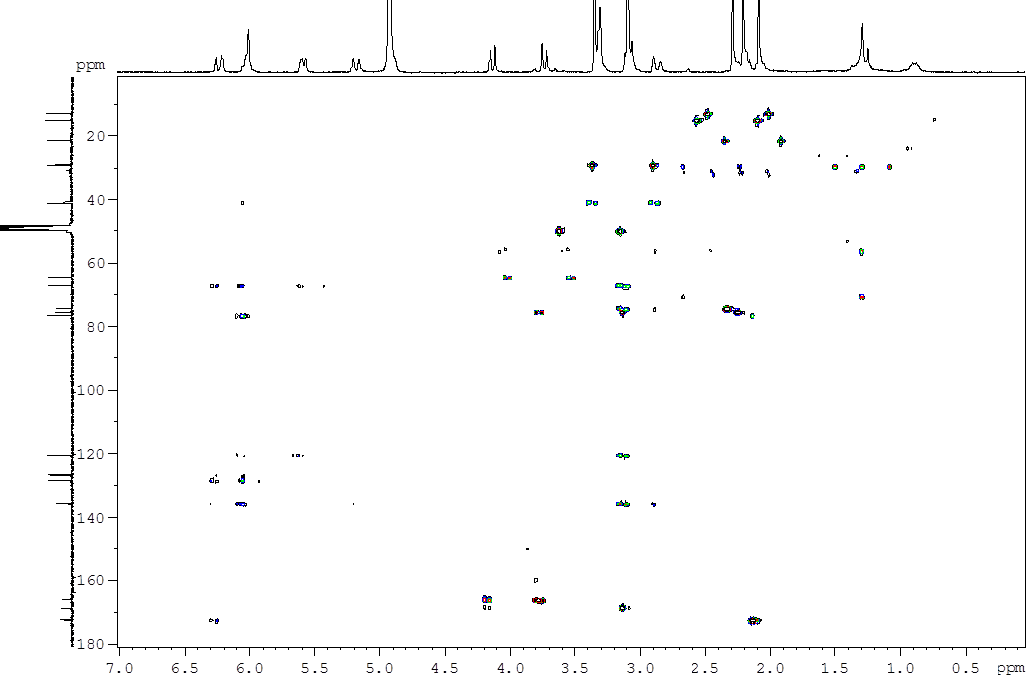
**

**Figure S2.8.** ^1^H-^13^C HMBC 2D-NMR spectrum of compound **2** in methanol-*d*_4_.

**
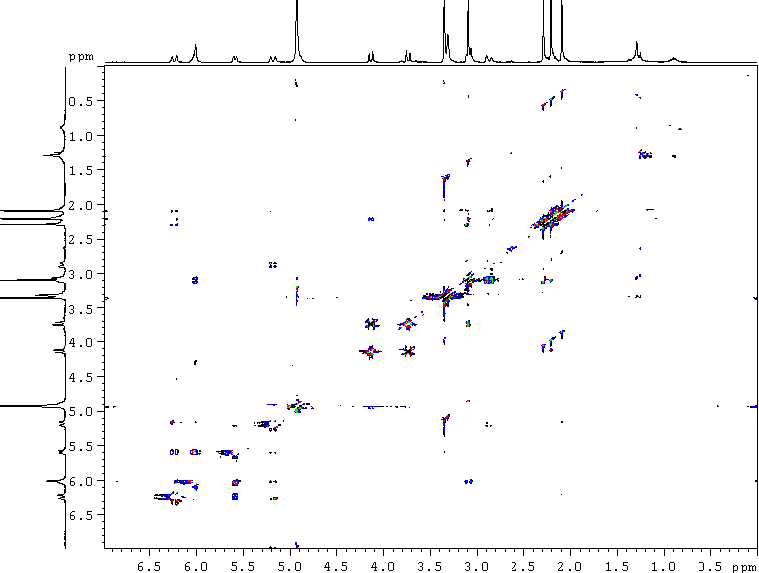
**

**Figure S2.9.** ^1^H-^1^H NOESY 2D-NMR spectrum of compound **2** in methanol-*d*_4_.


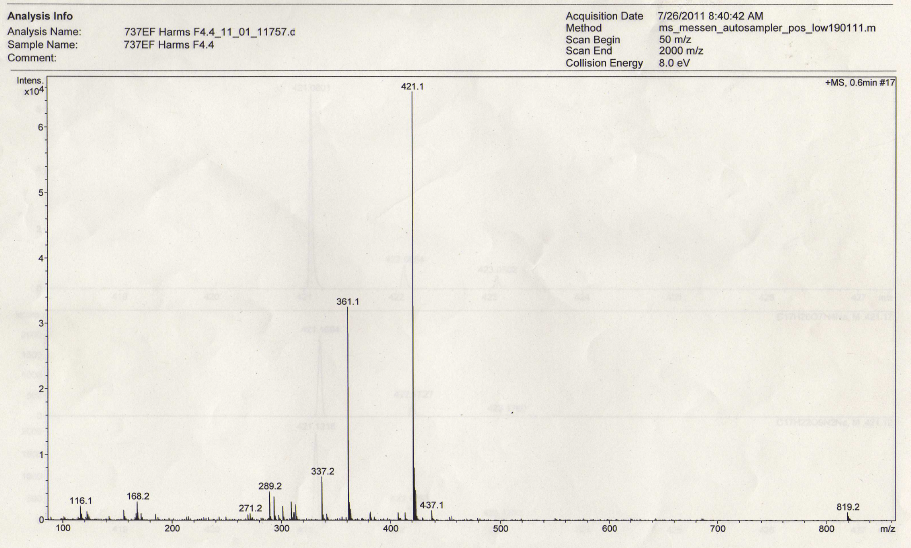


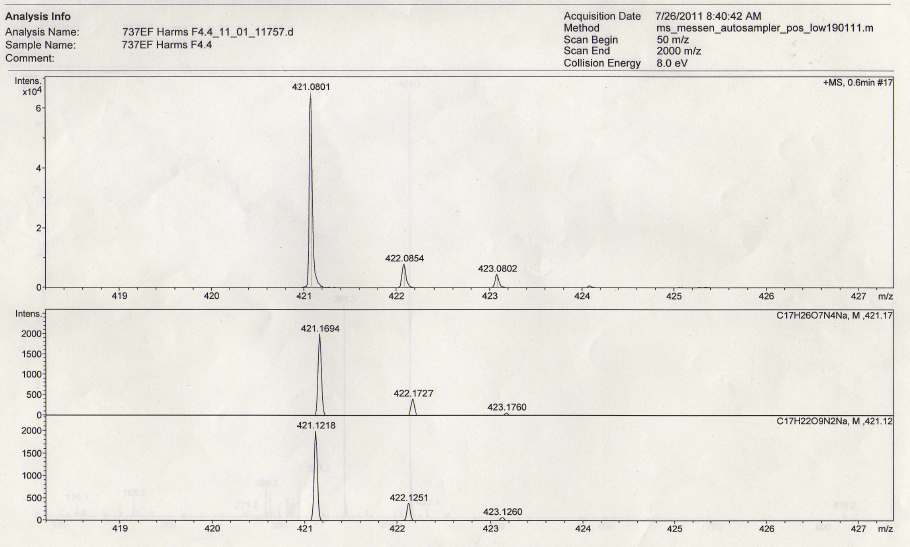


**Figure S2.10.** HRESIMS spectrum for compound **2**.


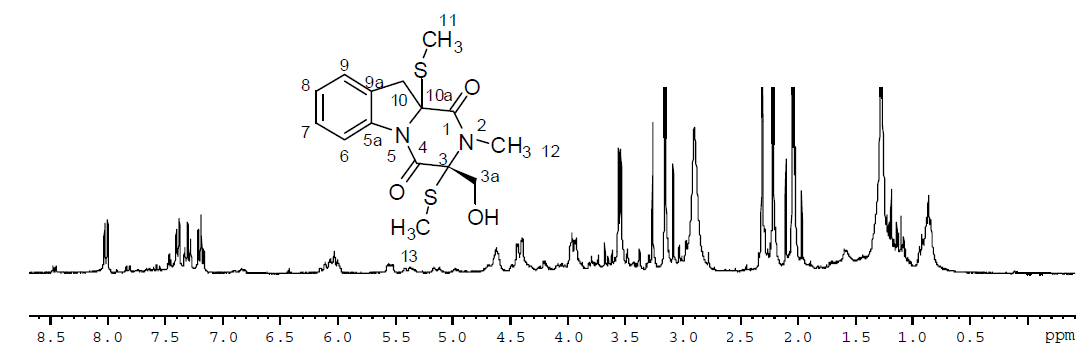


**Figure S3.1.** ^1^H NMR spectrum of compound **3** in acetone-*d*_6_.

**Figure S3.2.** ^13^C NMR spectrum of compound **3** in acetone-*d*_6_.

**Figure S3.3.** Dept 135 NMR spectrum of compound **3** in acetone-*d*_6_.

**Figure S3.4.** ^1^H-^13^C HSQC 2D-NMR spectrum of compound **3** in acetone-*d_6_*.

**Figure S3.5.** ^1^H-^1^H COSY 2D-NMR spectrum of compound **3** in acetone-*d*_6_.

**Figure S3.6.** ^1^H-^13^C HMBC 2D-NMR spectrum of compound **3** in acetone-*d*_6_.

**Figure S3.7.** ^1^H-^1^H NOESY 2D-NMR spectrum of compound **3** in acetone-*d*_6_.


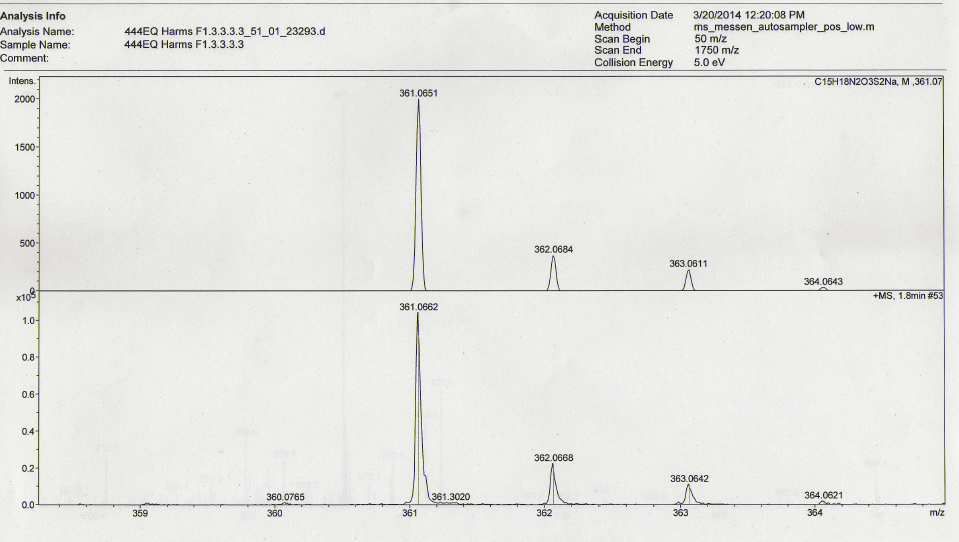


**Figure S3.8.** HRESIMS spectrum for compound **3**.

**Tables with spectroscopic data**

**Table S1.** NMR Spectroscopic Data (300 MHz, acetone-*d*_6_) for 6-acetylmonodethiogliotoxin (**1**).

| **Position** | **δ_C_, Mult** | **δ_H_ (J in Hz)** | **^1^H** -**^1^H COSY** | **^1^H**-**^13^C HMBC** | **^1^H** -**^1^H NOESY** |
| --- | --- | --- | --- | --- | --- |
| 1 | 174.2, C | - | - | - | - |
| 2 | N | - | - | - | - |
| 3 | 82.5, C | - | - | - | - |
| 3a | 58.0, CH_2_ | 3a′: 4.30, dd (5.9, 13.2)  3a″: 4.17, dd (5.9, 13.2) | 3a″  3a′ | 3, 4  3, 4 | 3a″, 5a  3a′, 6 |
| 4 | 171.3, C | - | - | - | - |
| 5 | N | - | - | - | - |
| 5a | 61.6, CH | 4.57, br d (13.5) | 6, 7, 8, 9, 10′, 10″ | 6, 9a | 3a′, 7, 8, 9, 10′, 13 |
| 6 | 74.9, CH | 5.79, br d (13.5) | 5a, 7, 8, 9 | 5a, 7, 8, 9, 9a, 10 | 3a″, 7, 8, 9, 10″, 13 |
| 7 | 127.1, CH | 5.53, br d (9.9) | 5a, 6, 8, 9 | 5a, 6, 8, 9 | 5a, 6, 8, 9 |
| 8 | 125.7 CH | 6.01, m | 5a, 6, 7 | 5a, 6, 7, 9, 9a, | 5a, 6, 7, 11 |
| 9 | 119.5 CH | 6.03, br s | 5a , 6, 7, 10′, 10″ | 5a, 6, 7, 8, 9a, 10 | 5a, 6, 7, 11 |
| 9a | 137.4, C | - | - | - | - |
| 10 | 29.2, CH_2_ | 10′: 3.45, d (18.3)  10″:2.98, d (18.3) | 5a, 9, 10″  5a. 9, 10′ | 5a, 1, 9, 9a, 10a  5a, 1, 9, 9a, 10a | 5a, 10″  6, 10′ |
| 10a | 80.4, C | - | - | - | - |
| 11 | 27.8, CH_3_ | 2.98, s | - | 1, 3 | 8, 9 |
| 12 | 170.5, C | - | - | - | - |
| 13 | 21.2, CH_3_ | 2.06, s | - | 3, 6 | 5a |
| 3a-OH | - | 4.74, br t (5.9) | 3a′, 3a″ | - | - |

**Table S2. NMR Spectroscopic Data (300 MHz, acetonel-*d_6_*) for 6-acetylbisdethiobis
(methylthio)gliotoxin (2).**

| **Position** | **δ_C_, Mult** | **δ_H_ (J in Hz)** | **^1^H** -**^1^H COSY** | **^1^H**-**^13^C HMBC** | **^1^H** -**^1^H NOESY** |
| --- | --- | --- | --- | --- | --- |
| 1 | 166.8, C | - | - | - | - |
| 2 | N | - | - | - | - |
| 3 | 74.5, C | - |  |  |  |
| 3a | 64.3, CH_2_ | 3a′: 4.17, dd (5.9, 11.3)  3a″: 3.72, dd (5.9, 11.3) | 3a″  3a′ | 3, 4  3, 4 | 3a″, 12, 13  3a′, 10′, 12 |
| 4 | 164.9, C | - | - | - | - |
| 5 | N | - | - | - | - |
| 5a | 66.3, CH | 5.10, br d (13.5) | 6, 7, 10′, 10″ | 9a | 7, 8, 9, 10″ |
| 6 | 75.7, CH | 6.17, br d (13.5) | 5a, 7, 8 | 5a, 7, 9, 10, 14, 15 | 7, 8, 9, 10′, 11, 13, 15 |
| 7 | 128.2, CH | 5.57, br d (9.9) | 6, 9, 10″ | 6, 9 | 5a, 6,9, 10′, 15 |
| 8 | 126.2, CH | 6.01, m | 5a, 6, 9, 10″ | 9 | 5a, 6, 11, 13 |
| 9 | 120.0, CH | 6.03, br s | 5a , 6, 7, 8, 10′, 10″ | 5a, 6, 7, 8, 9a, 10 | 5a, 6, 10′, 11, 13 |
| 9a | 135.8, C | - | - | - | - |
| 10 | 40.4, CH_2_ | 10′: 3.09, d (15.7)  10″: 2.79, d (15.7) | 5a, 10″  5a, 7, 9, 10′ | 5a, 9a, 10a′  5a, 9, 9a, 10a | 6, 7, 8, 9, 10″, 11  5a, 10′ |
| 10a | 73.6, C | - | - | - | - |
| 11 | 14.9, CH_3_ | 2.25, s | - | 10a | 6, 7, 9, 10′, 13 |
| 12 | 28.7, CH_3_ | 3.03, s | - | 1, 3, 3a | 3a′, 3a″, 13 |
| 13 | 12.7, CH_3_ | 2.18, s | - | 3 | 6, 7, 9, 10′, 11, 12, |
| 14 | 170.6, C | - | - | - | - |
| 15 | 21.3, CH_3_ | 2.02, s | - | 6, 14 | 6, 8 |
| 3a-OH | - | 4.50, br t (5.9) | 3a′, 3a″ | - | - |

**Table S3. NMR Spectroscopic Data (300 MHz, acetone-*d_6_*) for 5a,6-Anhydrobisdethiobis
(methylthio)gliotoxin (3).**

| **Position** | **δ_C_, Mult** | **δ_H_ (J in Hz)** | **^1^H-^1^H COSY** | **^1^H-^13^C HMBC** | **^1^H-^1^H NOESY** |
| --- | --- | --- | --- | --- | --- |
| 1 | 166.3, C | - | - | - | - |
| 2 | N | - | - | - | - |
| 3 | 73.2, C | - |  |  |  |
| 3a | 64.8, CH_2_ | 3a′: 4.42, dd (4.0, 12.1)  3a″: 3.95, dd (4.0, 12.1) | 3a″  3a′ | -  3, 4 | 3a″, 13  3a′, 12 |
| 4 | 162.9, C | - | - | - | - |
| 5 | N | - | - | - | - |
| 5a | 142.4, C | - | - | - | -‘ |
| 6 | 118.3, CH | 8.02, d (7.3) | 7 | 5a, 8, | 7 |
| 7 | 128.3, CH | 7.30, t (7.3) | 6, 8, 9 | 5a, 9a, 8, 9 | 8, 9 |
| 8 | 126.3, CH | 7.19, t (7.3) | 6, 7, 9 | 5a, 6, 7, 9, 9a | 7, 9 |
| 9 | 126.2, CH | 7.39, d (7.3) | 8, 10′, 10″ | 5a, 10 | 8, 10′ |
| 9a | 130.3, C | - | - | - | - |
| 10 | 40.0, CH_2_ | 10′: 3.59, d (16.8)  10″: 3.51, d (16.8) | 9, 10″  9, 10′ | 1, 5a, 9, 9a, 10a  1, 5a, 9, 9a, 10a | 9, 11 |
| 10a | 71.6, C | - | - | - | - |
| 11 | 14.4, CH_3_ | 2.22, s | - | 10a | 10′, 13 |
| 12 | 28.9, CH3 | 3.15, s | - | 1, 3, 3a | 3a′, 13 |
| 13 | 13.5, CH_3_ | 2.32, s | - | 3 | 11, 12, |
| 3a-OH | - | 4.62, br t (4.0) | 3a′, 3a″ | - | - |

**Purity data**

**Purity data for 6-acetylmonodethiogliotoxin (1):**

Purity of 6-acetylmonodethiogliotoxin was assessed with two different HPLC methods:

**Method A**: Waters HPLC system equipped with a 996 PDA detector (Waters GmbH, Hesse, Germany), a 600 pump (Waters GmbH, Hesse, Germany) and 717plus autosampler (Waters
GmbH, Hesse, Germany); column: Macherey-Nagel (MACHEREY-NAGEL GmbH & Co. KG, North-Rhine-Westphalia, Germany); EC 250 mm × 4.6 mm Nucleodur 100-5; RP_18_, 5 µm; mobile phase: acetonitrile–H_2_O (30–70); flow: 1.0 mL min^−1^.

**Method B**: Waters HPLC system equipped with a 996 PDA detector (Waters GmbH, Hesse, Germany), a 600 pump (Waters GmbH, Hesse, Germany) and 717 plus autosampler (Waters GmbH, Hesse, Germany); column: Waters (Waters GmbH, Hesse, Germany); 5 μm, EC 250 mm × 4.6 mm,
X-Terra (Waters GmbH, Hesse, Germany); RP_18_; mobile phase: MeOH–H_2_O (55–45); flow:
1.0 mL min^−1^.

**Table S4.** Purity analysis of 6-acetylmonodethiogliotoxin (**1**) with HPLC.

| **Method** | **Wavelength** | **Retention Time** | **Purity** |
| --- | --- | --- | --- |
| A | 265 nm | 11.05 min | 86.0% |
| A | 280 nm | 11.05 min | 86.1% |
| B | 250 nm | 14.47 min | 85.2% |
| B | 265 nm | 14.47 min | 93.7% |


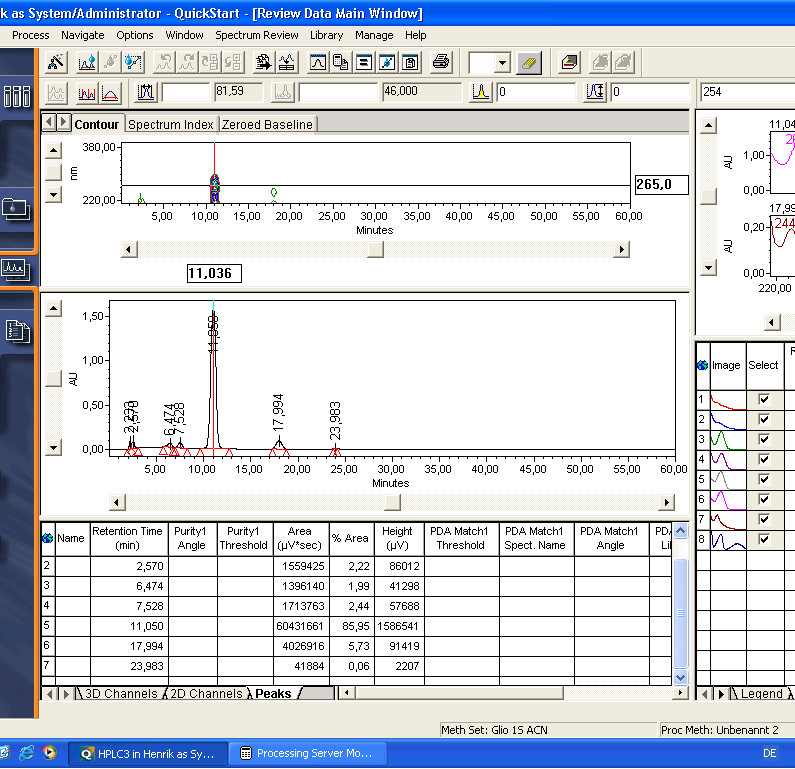


**Figure S4.1.** HPLC chromatogram of compound **1** using method A (265 nm).

**
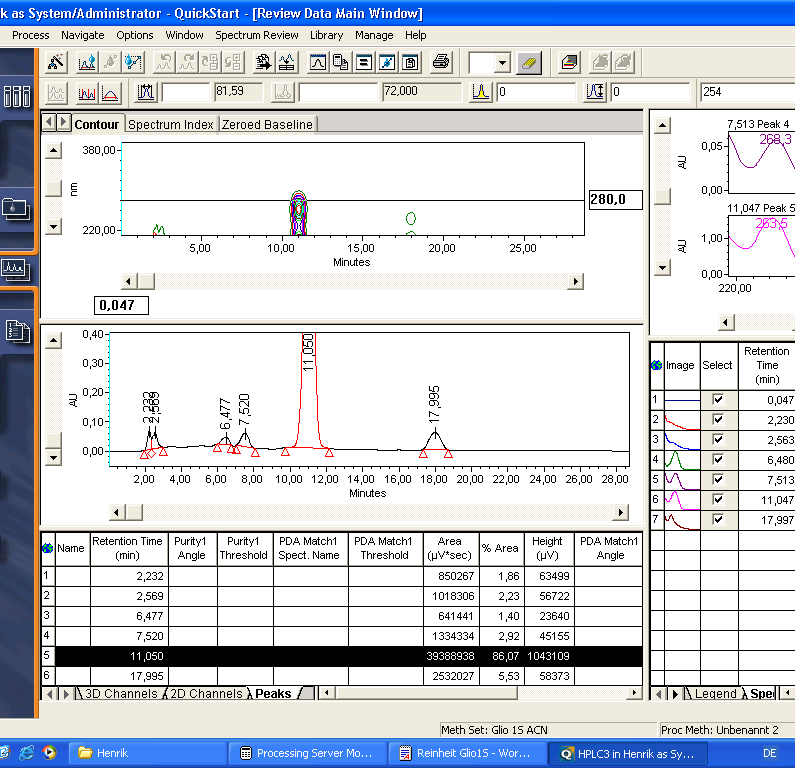
**

**Figure S4.2.** HPLC chromatogram of compound **1** using method B (280 nm).


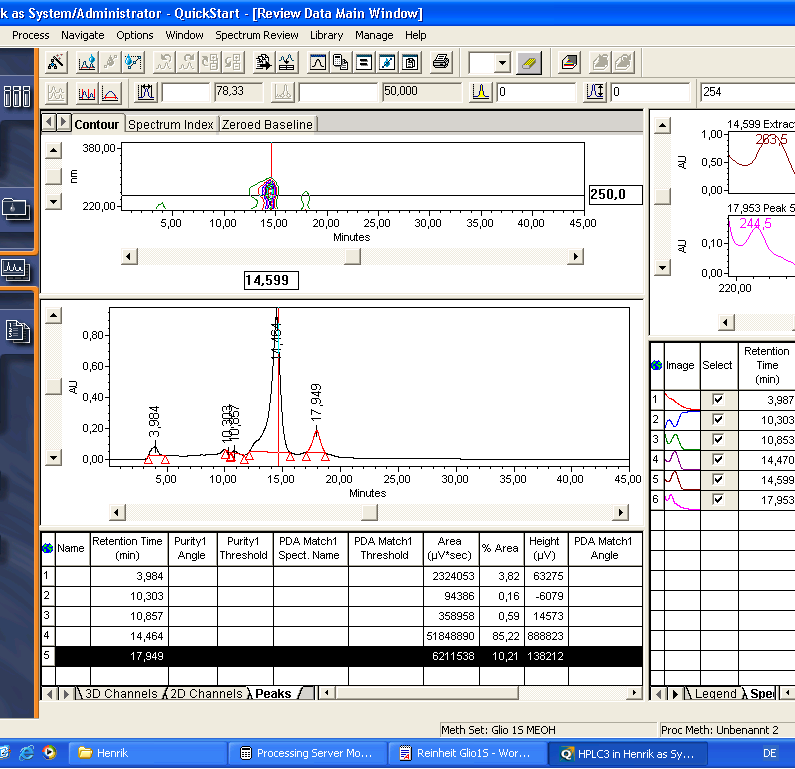


**Figure S4.3.** HPLC chromatogram of compound **1** using method B (250 nm).

**Figure S4.4.** HPLC chromatogram of compound **1** using method B (265 nm).

**Purity Data for 6-Acetylmonodethiogliotoxin (1) after Resolution:**

Purity of 6-acetylmonodethiogliotoxin after purification was assessed with the following HPLC method:

**Method C**:

Waters HPLC system equipped with a 996 PDA detector (Waters GmbH, Hesse, Germany),
a 600 pump and 717plus autosampler (Waters GmbH, Hesse, Germany; column: Waters (Waters GmbH, Hesse, Germany); 5 μm, EC 250 mm × 4.6 mm, X-Terra (Waters GmbH, Hesse, Germany); RP_18_; mobile phase: MeOH–H_2_O (50–50); flow: 0.6 mL min^−1^.

**Table S5.** Purity analysis of 6-acetylmonodethiogliotoxin (**1**) with HPLC after reisolation.

| **Method** | **Wavelength** | **Retention Time** | **Purity** |
| --- | --- | --- | --- |
| C | 265 nm | 10.2 min | 99.6% |
| C | 240 nm | 10.2 min | 97.6% |


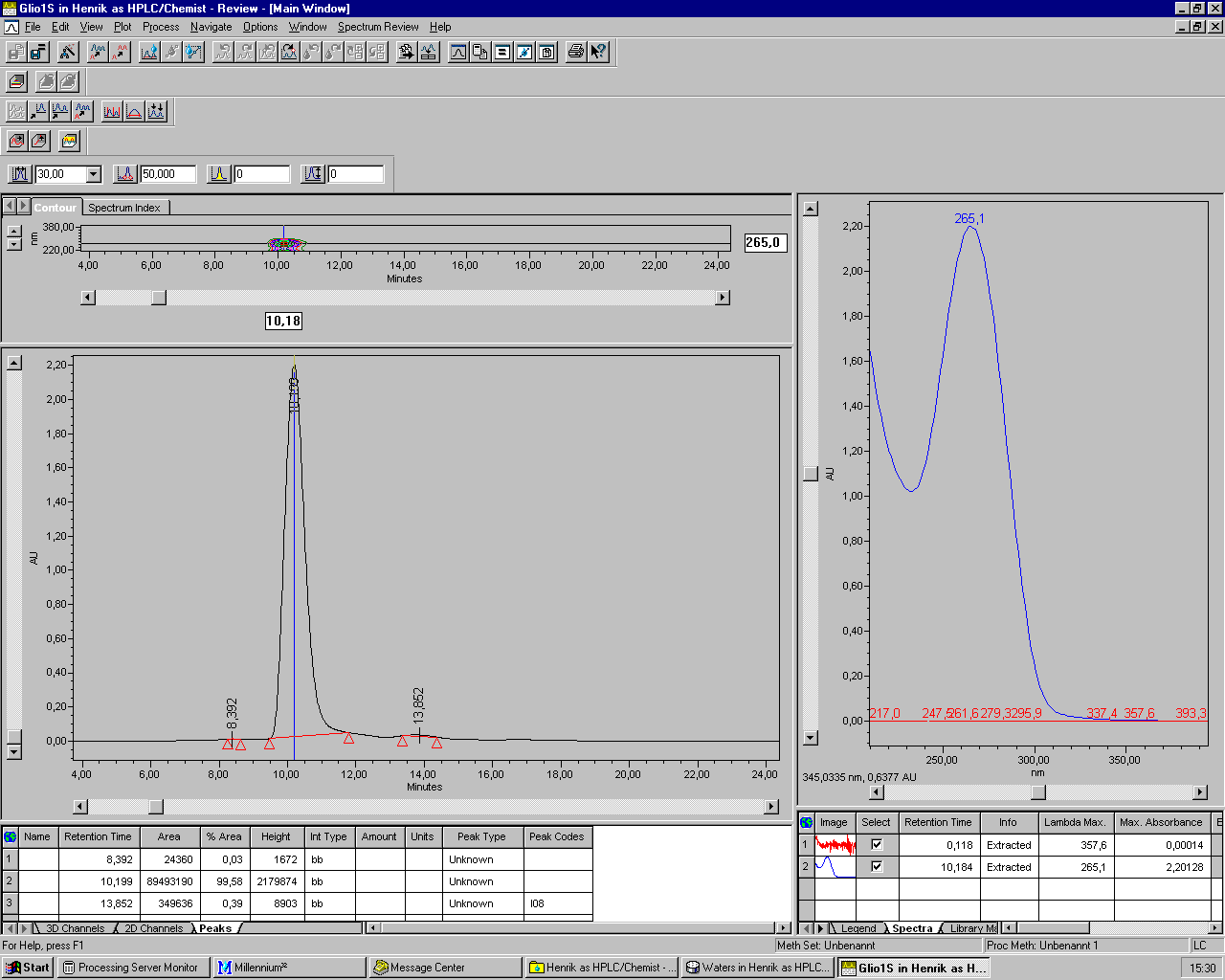


**Figure S4.5.** HPLC chromatogram of compound **1** using method C after reisolation (265 nm).


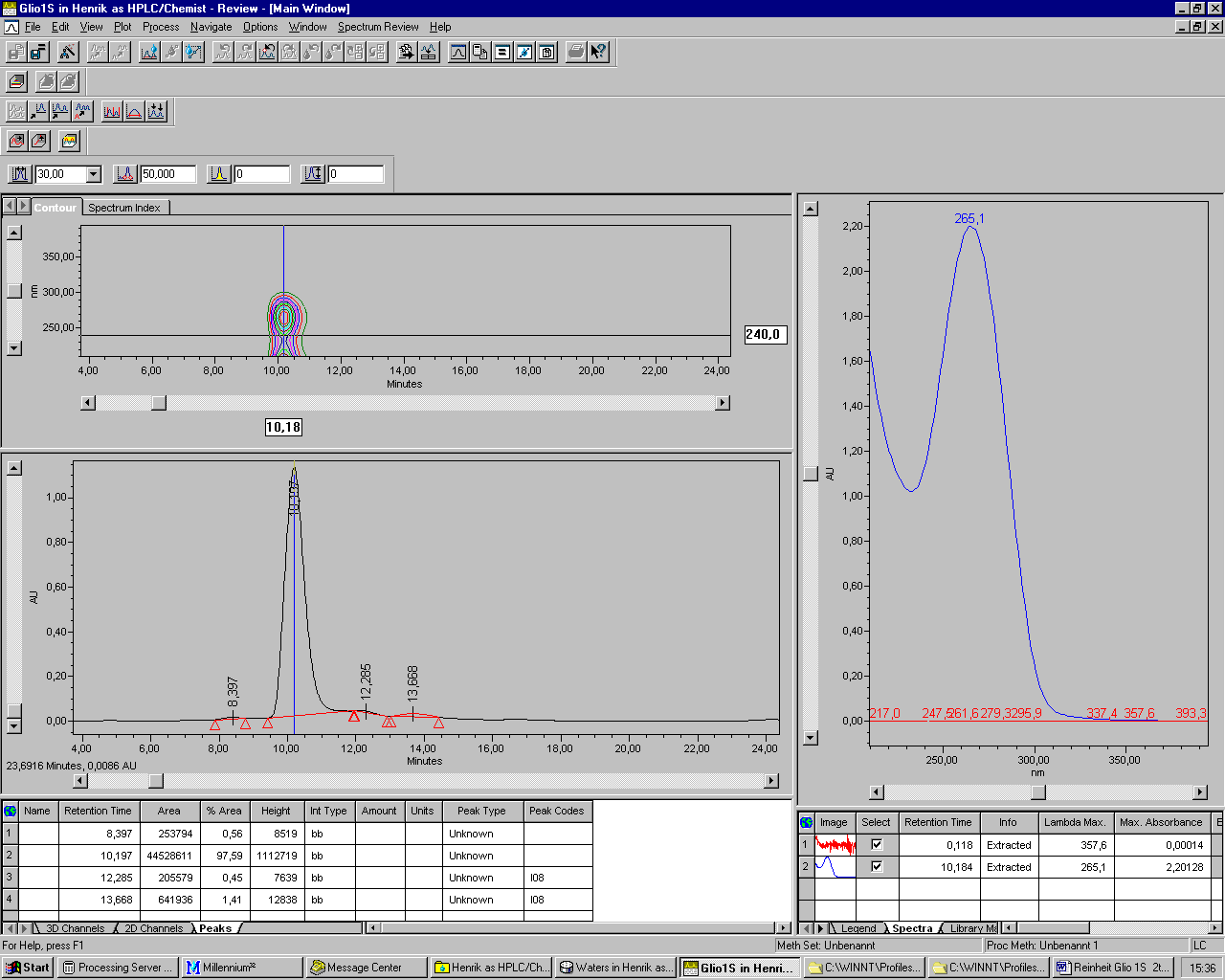


**Figure S4.6.** HPLC chromatogram of compound **1** using method B (240 nm).

Purity data for 6-acetylbisdethiobis(methylthio)gliotoxin (2):

Purity of 6-acetylbisdethiobis(methylthio)gliotoxin was assessed with two different HPLC methods:

**Method A**:

Waters HPLC system equipped with a 996 PDA detector (Waters GmbH, Hesse, Germany),
a 600 pump and 717plus autosampler (Waters GmbH, Hesse, Germany); column: Macherey-Nagel (MACHEREY-NAGEL GmbH & Co. KG, North-Rhine-Westphalia, Germany), 5 µm, EC
250 mm × 4.6 mm; Nucleodur 100-5; RP_18_, mobile phase: MeOH–H_2_O (62–38) flow: 0.6 mL min^−1^.

**Method B**:

Waters HPLC system equipped with a 996 PDA detector (Waters GmbH, Hesse, Germany), a 600 pump and 717plus autosampler (Waters GmbH, Hesse, Germany); column: Waters (Waters GmbH, Hesse, Germany), 5 μm, EC 250 mm × 4.6 mm, Atlantis, RP_18_, mobile phase: acetonitrile–H_2_O
(25–75); flow: 1.0 mL min^−1^.

**Table S6.** Purity analysis of 6-acetylbisdethiobis(methylthio)gliotoxin (**2**) with HPLC.

| **Method** | **Wavelength** | **Retention Time** | **Purity** |
| --- | --- | --- | --- |
| A | 250 nm | 12.49 min | 95.25% |
| A | 280 nm | 12.49 min | 91.52% |
| B | 250 nm | 25.82 min | 97.64% |
| B | 280 nm | 25.82 min | 99.20% |


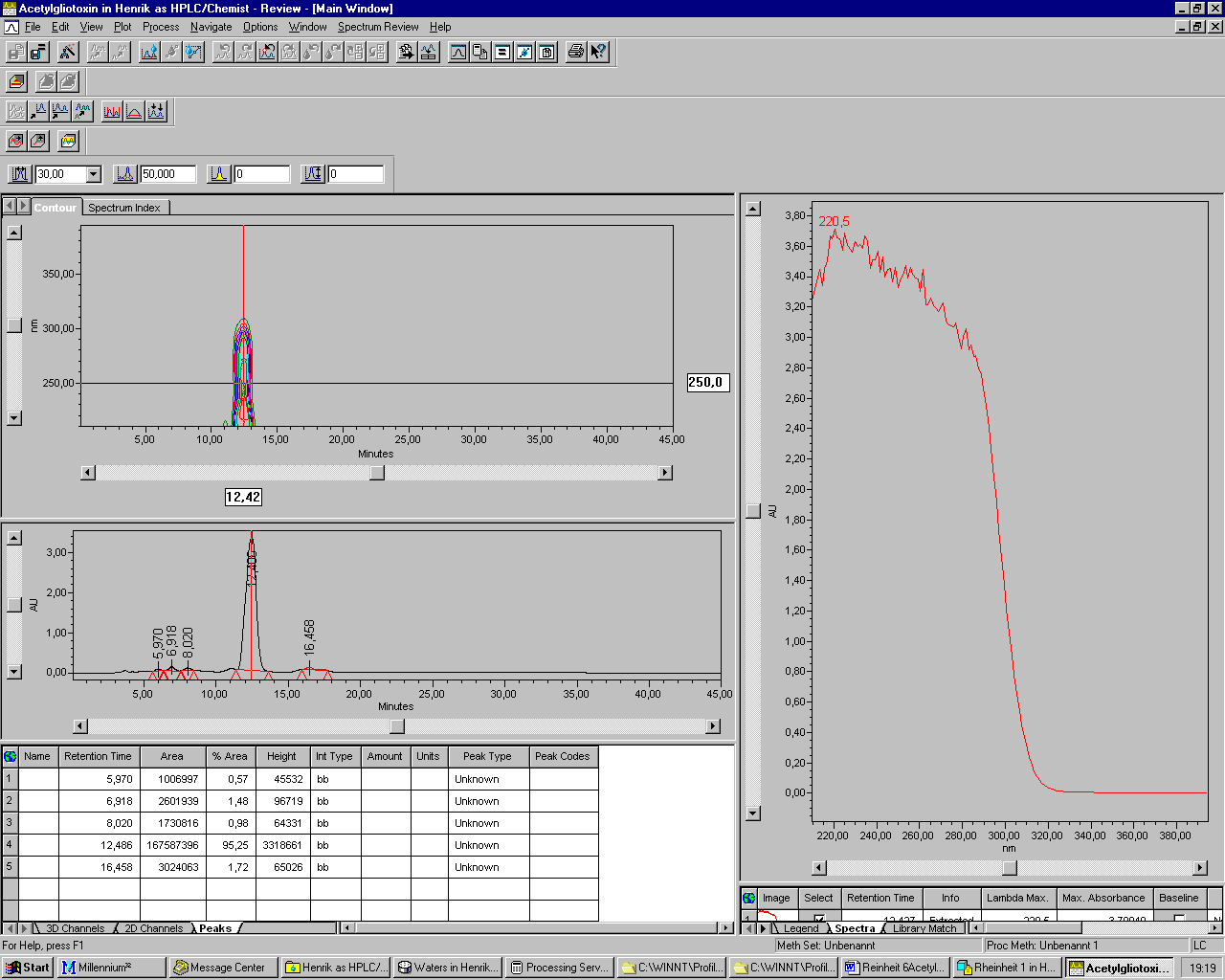


**Figure S5.1.** HPLC chromatogram of compound **2** using method A (250 nm).


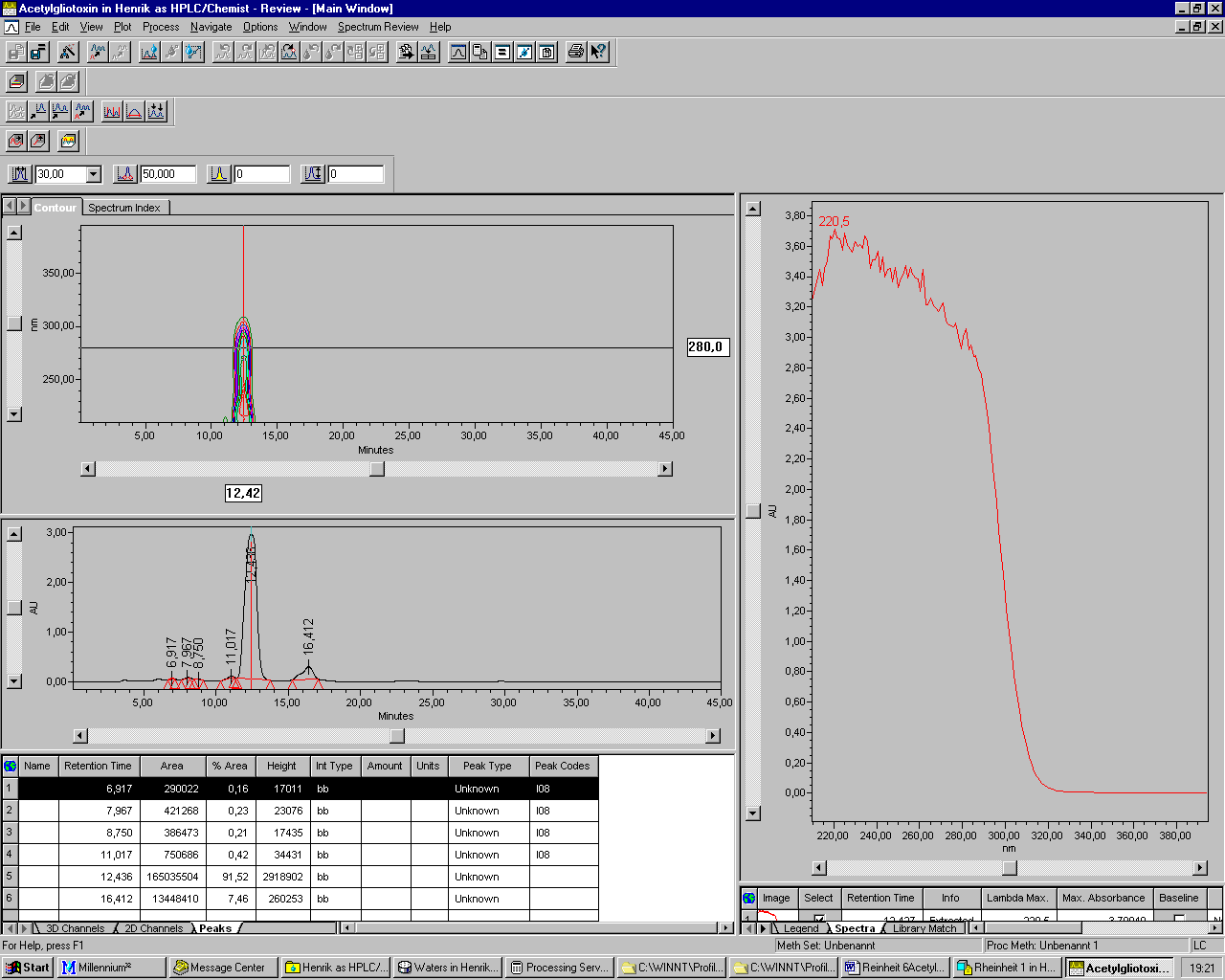


**Figure S5.2.** HPLC chromatogram of compound **2** using method A (280 nm).


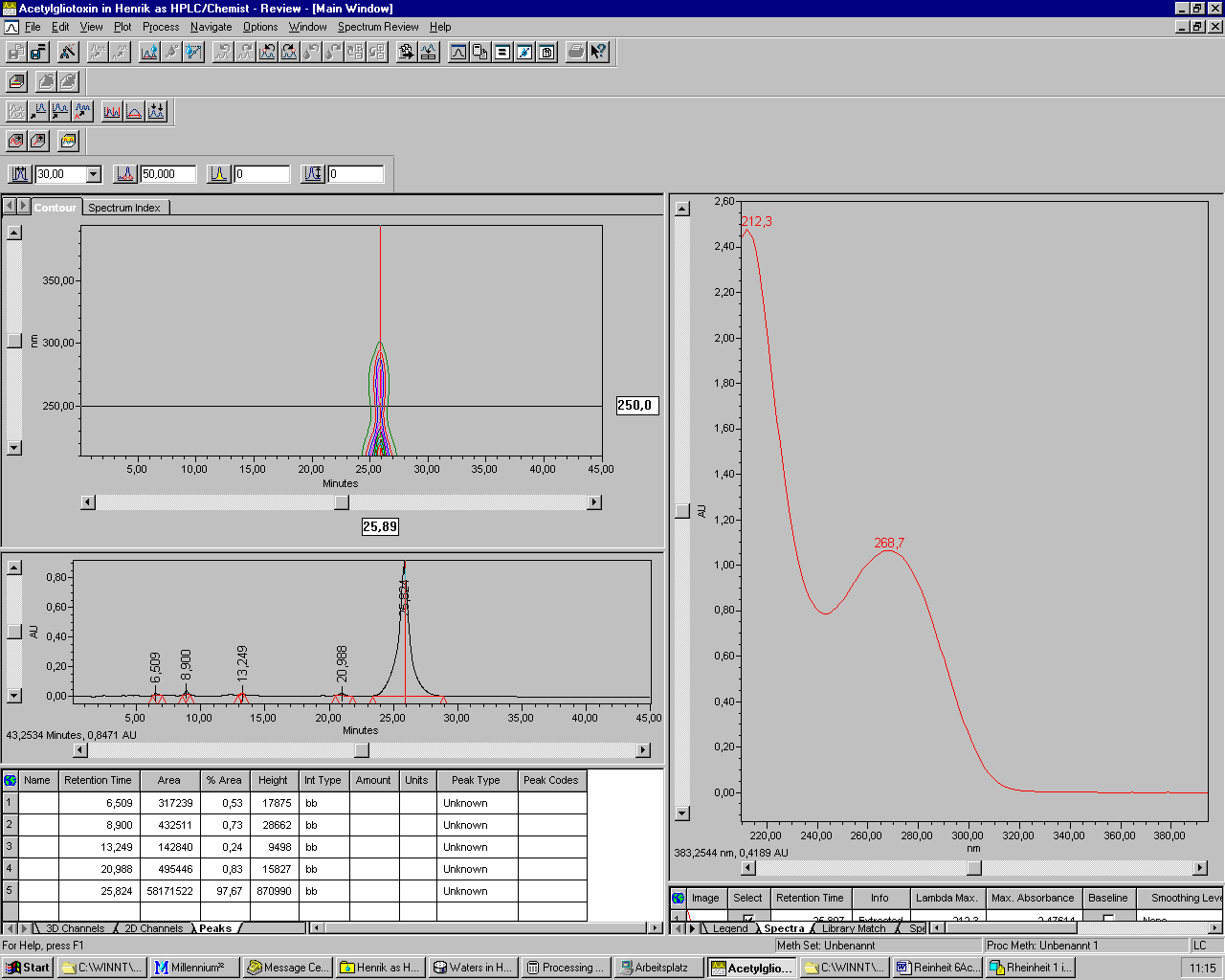


**Figure S5.3.** HPLC chromatogram of compound **2** using method B (250 nm).


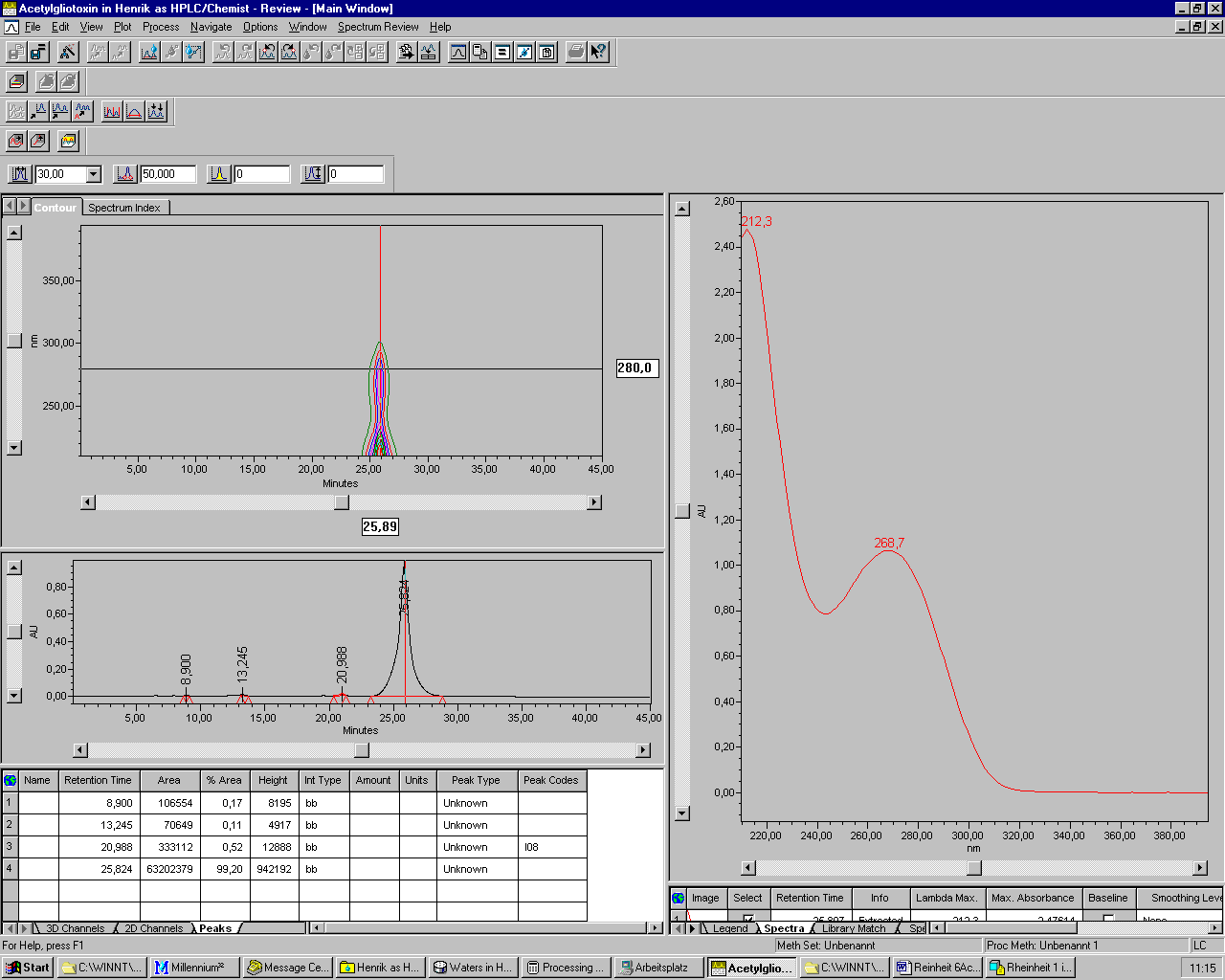


**Figure S5.4.** HPLC chromatogram of compound **2** using method B (280 nm).

**Purity data for heveadride (4):**

Purity of heveadride was assessed with HPLC:

**Method A**:

Waters HPLC system equipped with a 996 PDA detector (Waters GmbH, Hesse, Germany), a 600 pump and 717plus autosampler (Waters GmbH, Hesse, Germany); column: Knauer (KNAUER Wissenschaftliche Geräte GmbH, Berlin, Germany), 5 µm, EC 250 mm × 8.0 mm, Eurospher II 100-5, RP-C_8_, mobile phase: CAN–H_2_O (95–05); flow: 1.0 mL min^−1^.

**Method B**:

Waters HPLC system equipped with a 996 PDA detector (Waters GmbH, Hesse, Germany), a 600 pump and 717plus autosampler (Waters GmbH, Hesse, Germany); column: Waters (Waters GmbH, Hesse, Germany) Atlantis RP_18_, 5 μm, EC 250 mm × 4.6 mm; mobile phase: acetonitrile–H_2_O (50–50); flow: 1.0 mL min^−1^.

**Table S7.** Purity analysis of heveadride (**4**) with HPLC.

| **Method** | **Wavelength** | **Retention Time** | **Purity** |
| --- | --- | --- | --- |
| A | 240 nm | 10.16 min | 99.8% |
| A | 270 nm | 10.16 min | 97.4% |
| B | 240 nm | 34.20 min | 99.7% |
| B | 270 nm | 34.20 min | 99.5% |


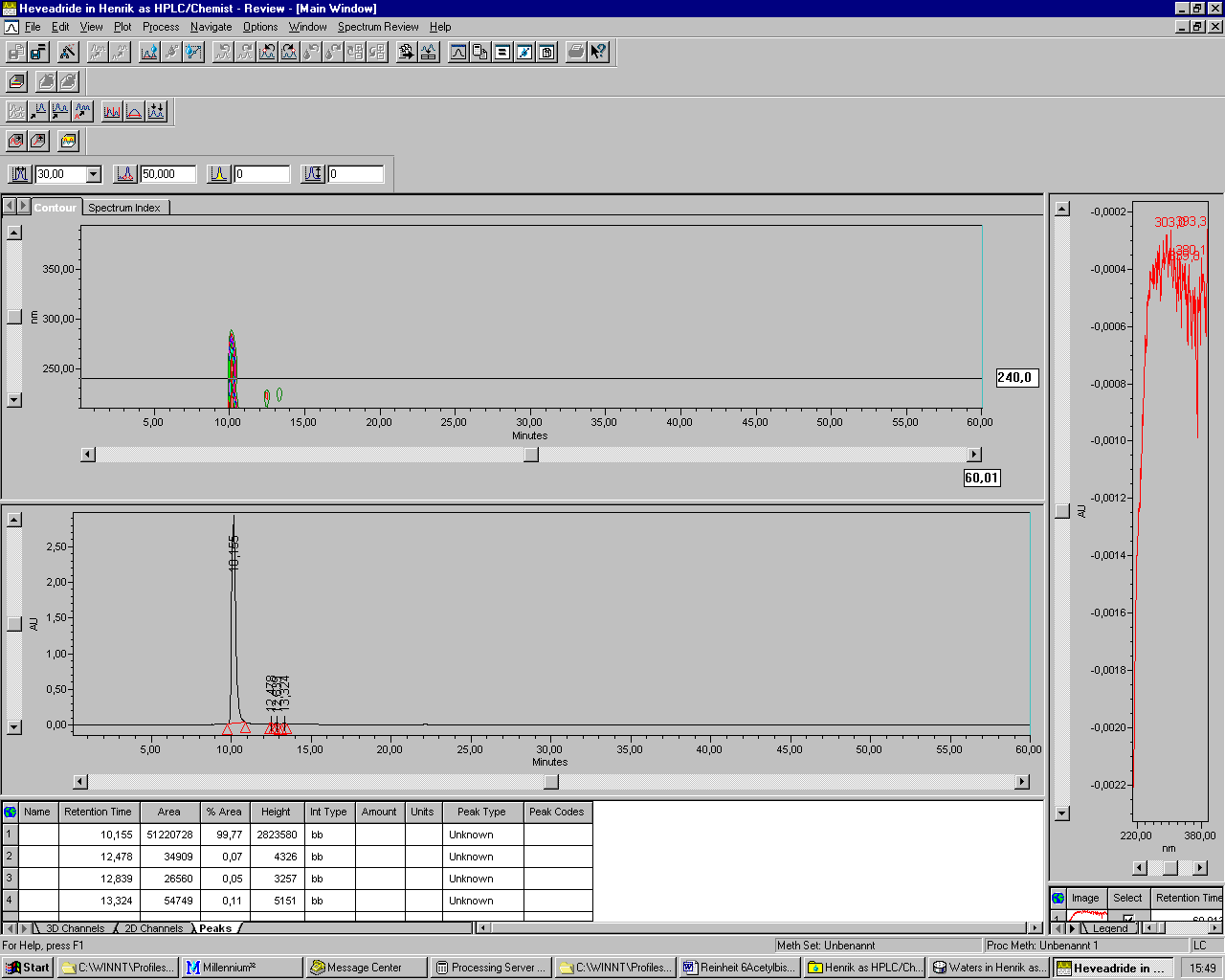


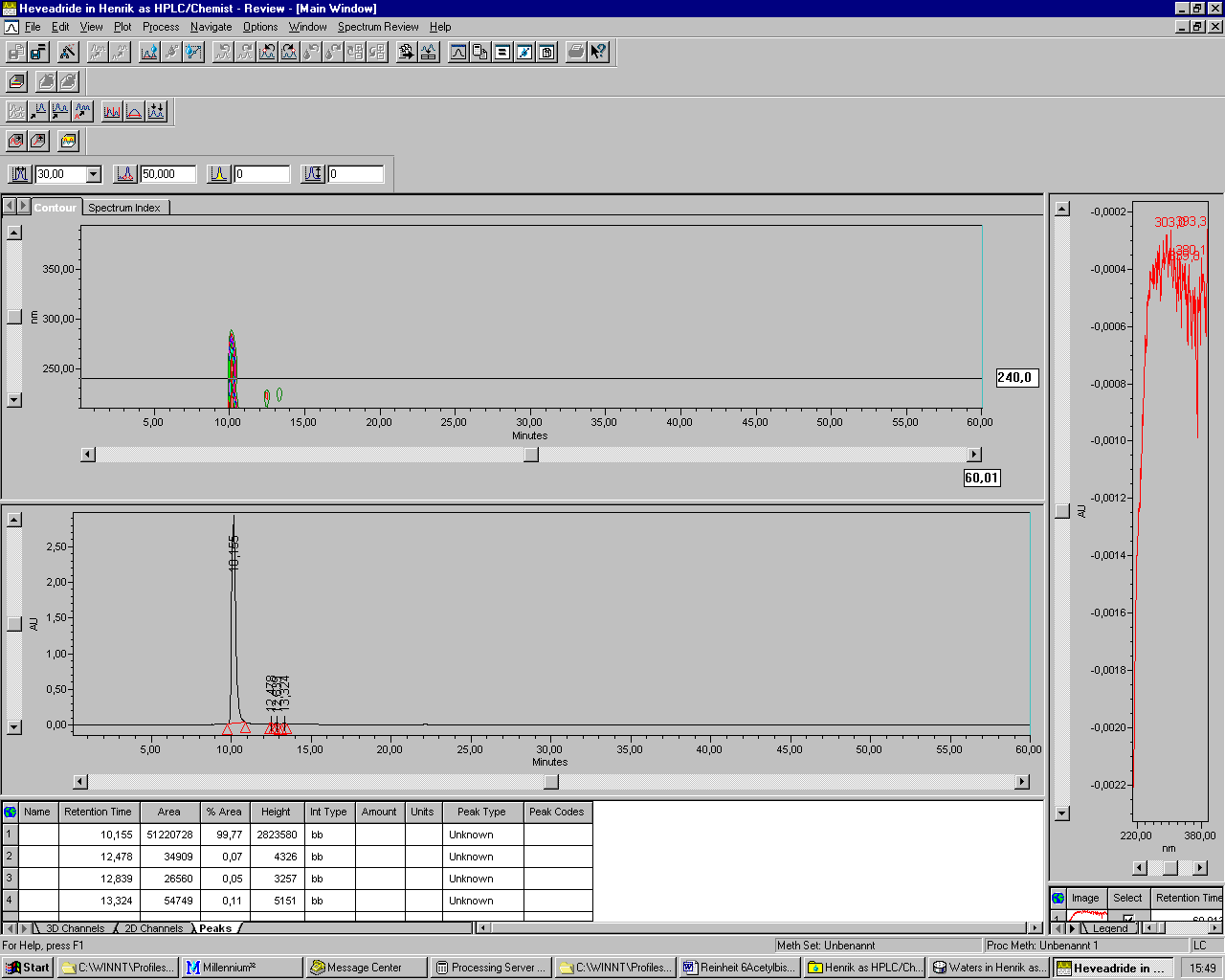


**Figure S6.1.** HPLC chromatogram of compound **4** using method A (240 nm).


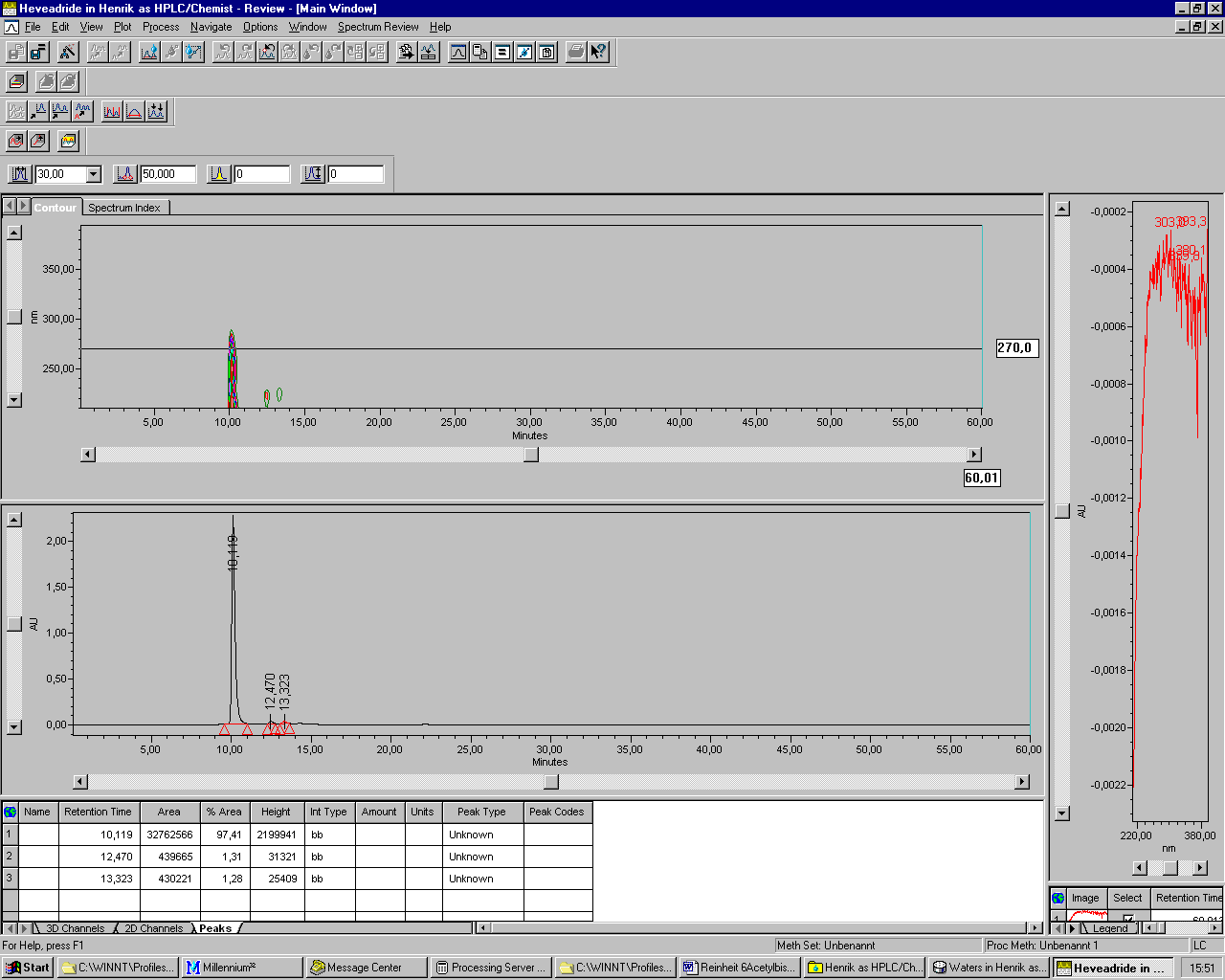


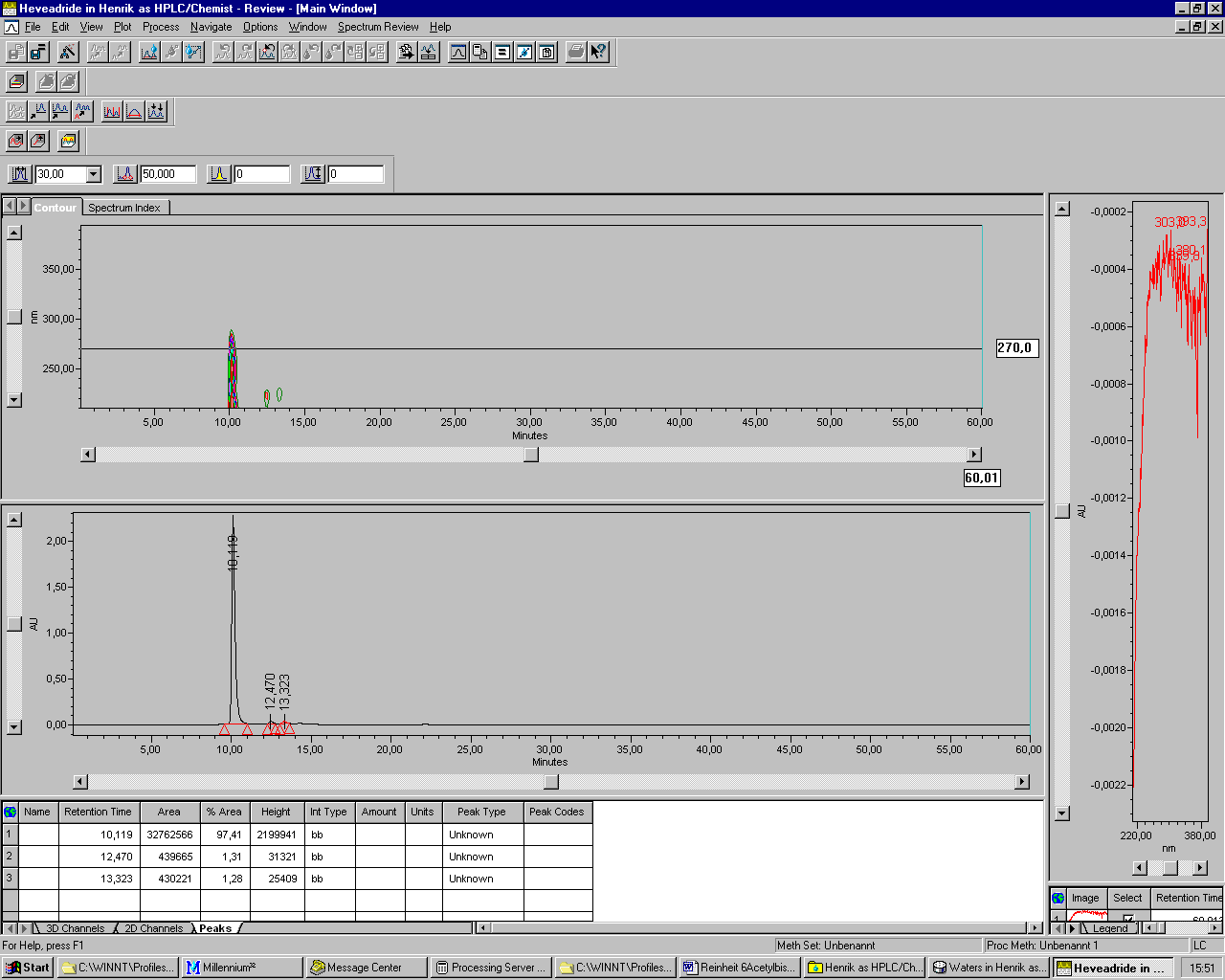


**Figure S6.2.** HPLC chromatogram of compound **4** using method A (270 nm).


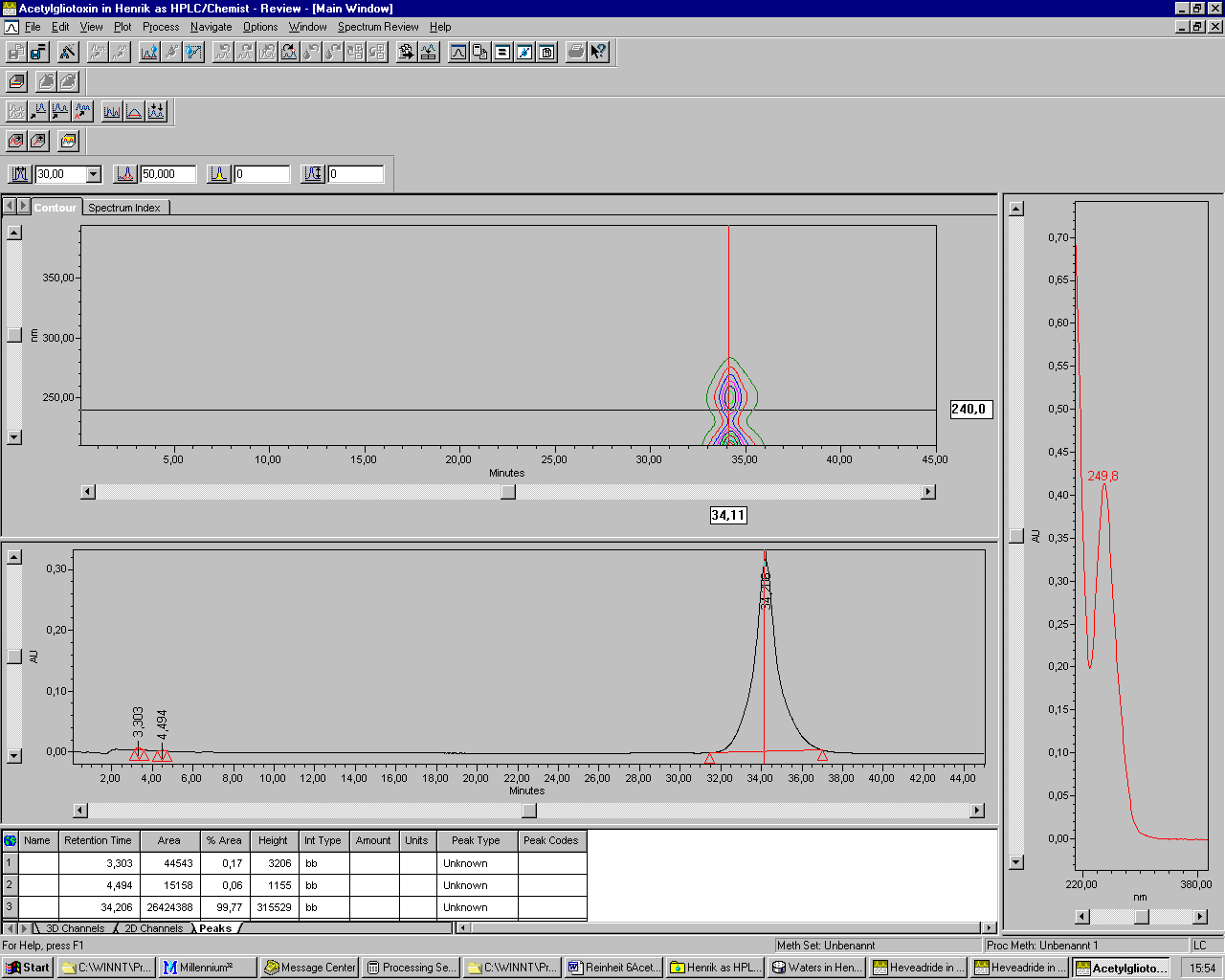


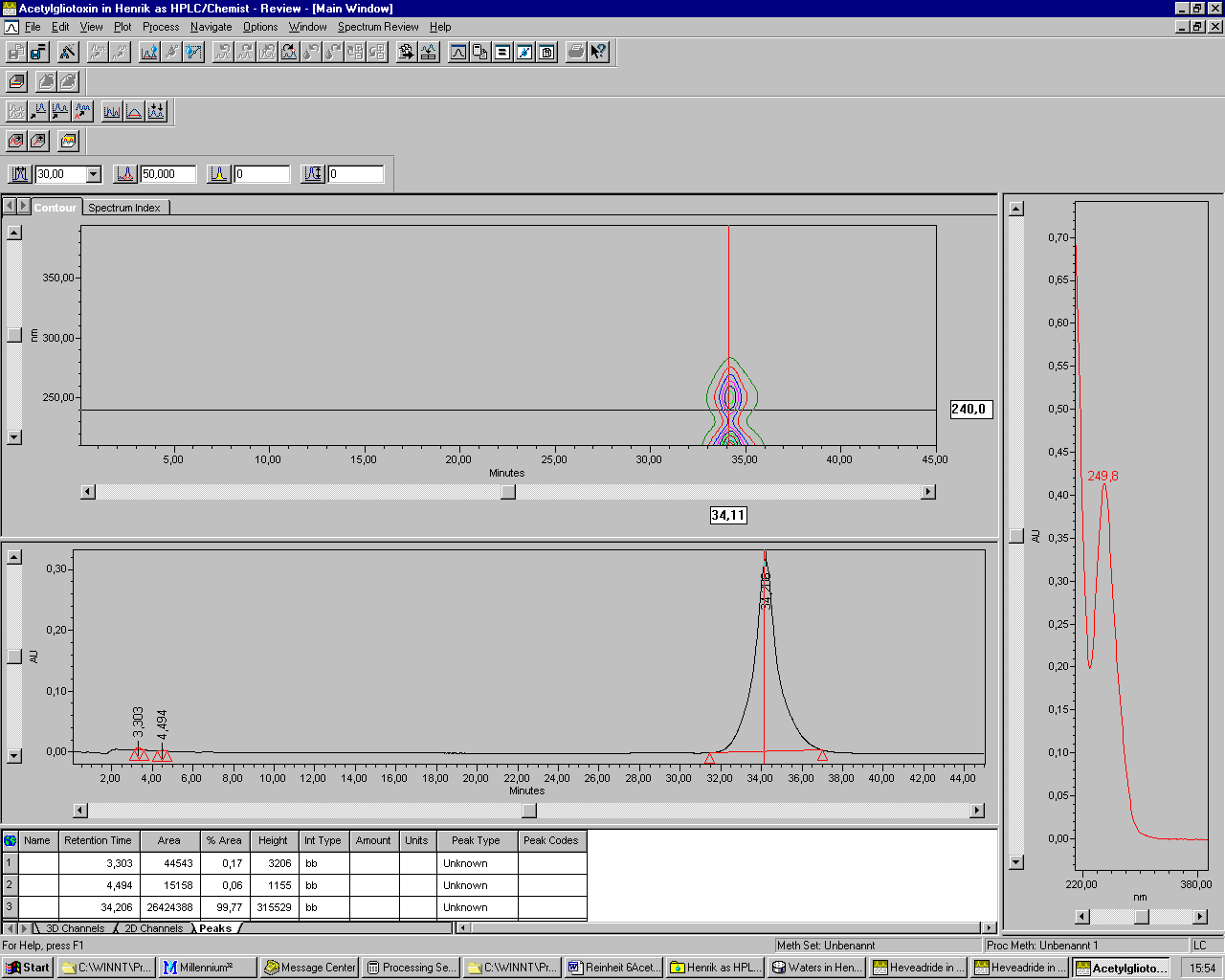


**Figure S6.3.** HPLC chromatogram of compound **4** using method A (240 nm).


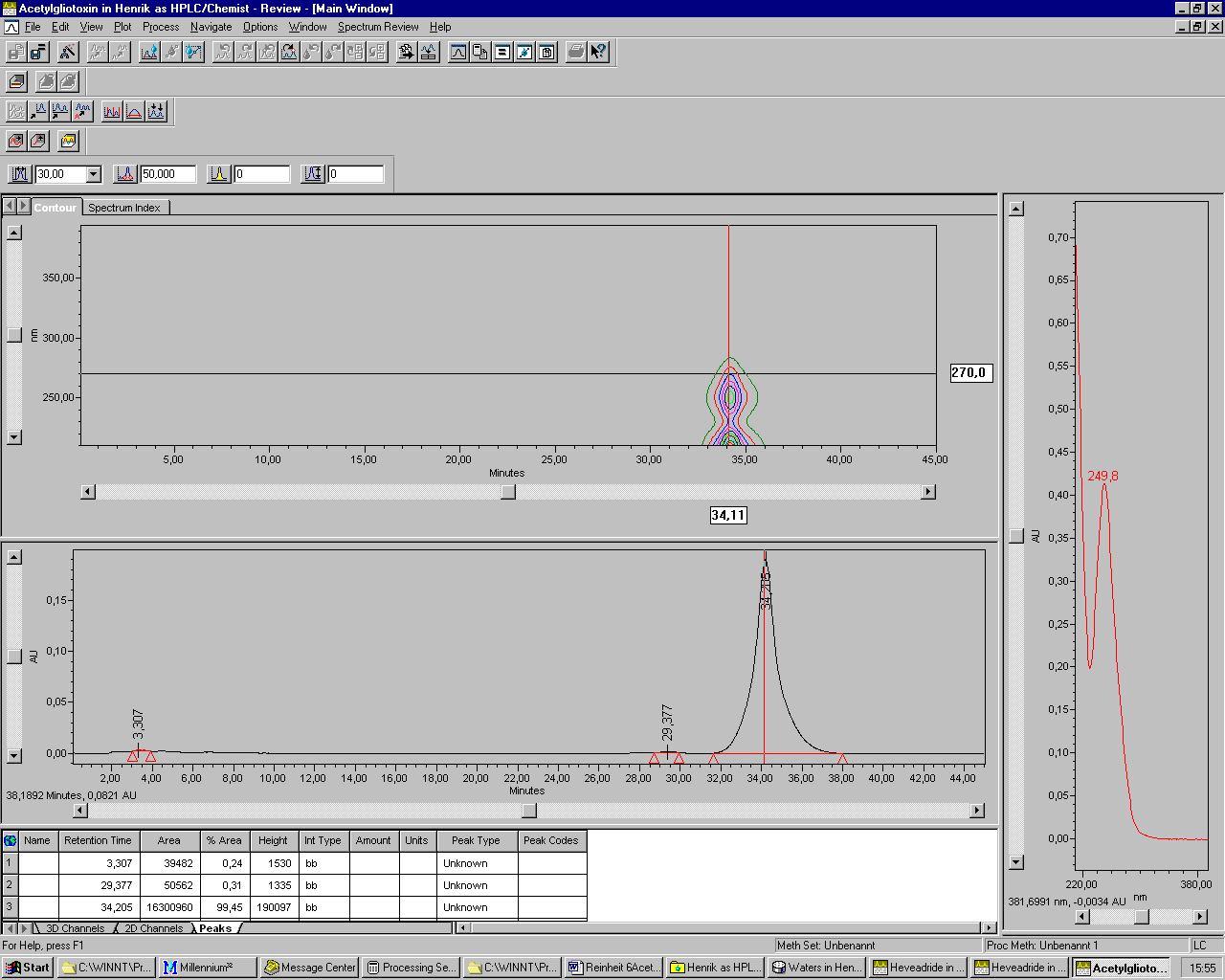


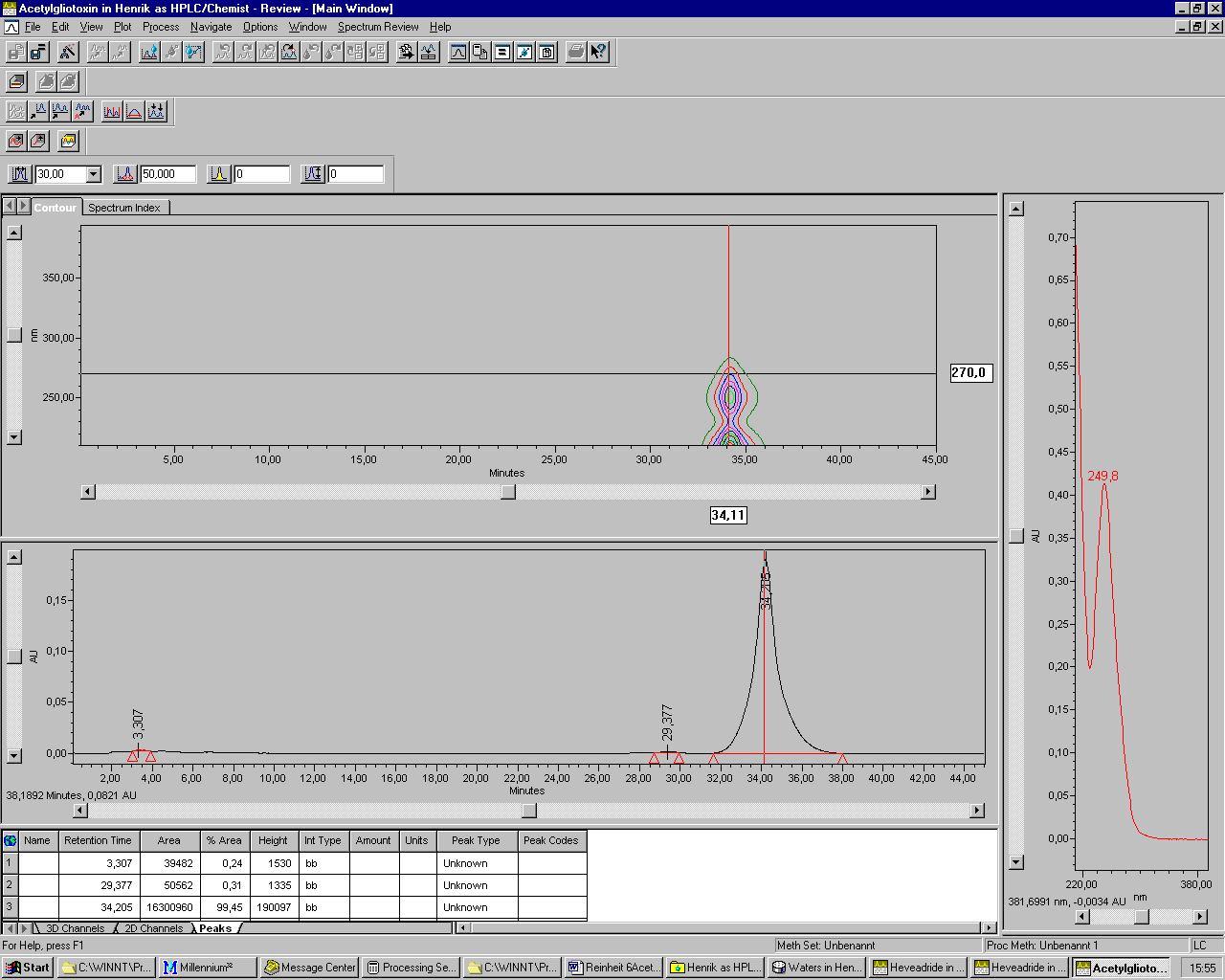


**Figure S6.4.** HPLC chromatogram of compound **4** using method A (270 nm).

**NMR Key correlations**

**Figure S7.1.** Key ^1^H-^1^H COSY and ^1^H-^13^C HMBC correlations of compound **1**.

**Figure S7.2.** Key ^1^H-^1^H NOESY correlations of compound **1**.

**Figure S7.3.** Key ^1^H-^1^H COSY and ^1^H-^13^C HMBC correlations of compound **2**.

**Figure S7.4.** Key ^1^H-^1^H NOESY correlations of compound **2**.


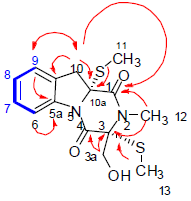


**Figure S7.5.** Key ^1^H-^1^H COSY and ^1^H-^13^C HMBC correlations of compound **3**.

**
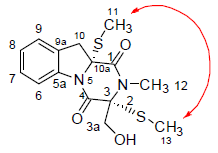
**

**Figure S7.6.** Key ^1^H-^1^H NOESY correlations of compound **3**.

© 2015 by the authors; licensee MDPI, Basel, Switzerland. This article is an open access article distributed under the terms and conditions of the Creative Commons Attribution license (http://creativecommons.org/licenses/by/4.0/).
